# Optimized planting density and nitrogen improve grain yield and water productivity in drip-fertigated maize through improved canopy function and source–sink balance

**DOI:** 10.3389/fpls.2026.1740500

**Published:** 2026-02-19

**Authors:** Zhenlin Lai, Zhenqi Liao, Hao Kong, Yiyao Liu, Hongtai Kou, Kechun Wang, Zhijun Li, Junliang Fan

**Affiliations:** Key Laboratory of Agricultural Soil and Water Engineering in Arid and Semiarid Areas of Ministry of Education, Northwest Agriculture and Forestry (A&F) University, Xianyang, Shaanxi, China

**Keywords:** drip-fertigated maize, grain yield, post-silking leaf functional decline, source-sink relation, water productivity

## Abstract

**Introduction:**

Balanced source–sink relations are essential for achieving high maize yield and water productivity, and maintaining post-silking green leaf area is critical for dry matter accumulation and yield formation in maize (*Zea mays* L.). However, the mechanisms by which nitrogen (N) rate and planting density affect yield formation via leaf senescence and source–sink regulation remain unclear. This study aimed to elucidate the respective contributions of post-silking leaf functional decline and source–sink balance to grain yield and water productivity of drip-irrigated maize.

**Methods:**

A two-year field experiment was conducted in northwest China with three planting densities (LD: 80,000; MD: 100,000; HD: 120,000 plants ha^-1^) and four N rates (N0: 0; N1: 120; N2: 180; N3: 240 kg N ha^-1^). Leaf area duration (LAD), post-silking leaf functional decline, source–sink traits, grain yield, and water productivity were evaluated, and relationships among key variables were analyzed using PLS-SEM.

**Results:**

Nitrogen application alleviated stress-induced premature leaf functional decline after silking, whereas increasing planting density accelerated the loss of effective leaf function. Both higher planting density and higher N rate significantly increased LAD. Compared with LD, MD and HD increased source growth by 23.0% and 19.4%, sink capacity by 23.9% and 15.2%, sink growth rate by 23.7% and 15.8%, and source–sink difference by 16.2% and 18.2%, respectively, indicating that sink limitation constrained further yield increases at higher densities. PLS-SEM showed that N mitigated premature leaf functional decline, while planting density negatively affected leaf functional maintenance. Planting density indirectly affected LAD via leaf functional decline (63.7% of the effect), whereas N rate and planting density directly influenced LAD (75.6% of the effect). LAD strongly affected source growth parameters (90.7%), which increased grain yield (89.0%) and source–sink parameters (73.4%), ultimately contributing to direct increases in grain yield (81.8%) and water productivity (74.3%). D2N3 achieved the highest grain yield, followed by D2N2, which significantly improved water productivity and irrigation water productivity.

**Discussion:**

Considering source–sink balance, water saving, and stable yield, D2N2 is recommended. These results improve understanding of how to achieve effective dense planting and high-yield maize cultivation under water-scarce conditions.

## 1 Introduction

Maize (Zea mays L.) is a critical staple crop, yet its yield potential is frequently constrained by physiological limitations imposed by environmental and management factors. While increasing planting density is a primary strategy to enhance productivity by optimizing population structure and light interception (Li et al., 2021), it simultaneously induces crowding stress. This stress diminishes leaf photosynthetic capacity, accelerates leaf senescence, and alters the source-sink balance, ultimately limiting individual plant performance (Li et al., 2015). Nitrogen (N) availability is fundamental in modulating these physiological responses; it regulates phenological development, leaf expansion, and the maintenance of photosynthetic duration (Chen et al., 2015; Ning et al., 2017). In the arid and semi-arid regions of Northwest China, crops face the dual challenges of crowding stress and chronic water scarcity (Xu et al., 2021). Under these conditions, the physiological mechanisms by which maize plants coordinate carbon assimilation and partitioning under varying N supplies and densities remain not fully understood. Therefore, rather than simply identifying high-yield agronomic practices, it is essential to elucidate the physiological basis of how N modulates the trade-off between crowding stress and resource use efficiency to overcome yield limitations.

The source-sink relationship is the central physiological process governing dry matter accumulation and partitioning (Mason and Maskell, 1928; Ma et al., 2025). The “source” represents the potential photosynthetic capacity, primarily in leaves, while the “sink” denotes the capacity to store assimilates, represented by kernels in maize (Borrás et al., 2004; Li et al., 2022). Yield formation depends on the coordination between source activity and sink strength (Causin et al., 2006). Although increasing planting density theoretically enhances population-level source and sink capacity, it often creates a source-sink imbalance at the individual plant level due to accelerated senescence and reduced assimilation per plant (He et al., 2002). Nitrogen plays a pivotal role in regulating this source-sink dynamics, influencing sink formation and the mass transfer of assimilates (Distelfeld et al., 2014). Severe N stress restricts both sink capacity and source productivity (Ordóñez et al., 2018), whereas optimized N supply can maximize source-sink capacity (Wu et al., 2022). However, the specific physiological mechanisms by which N availability mitigates the negative effects of high-density crowding stress on source-sink equilibration remain unclear.

A critical, yet under-evaluated component of this relationship is the pattern of post-silking leaf senescence (Abeledo et al., 2020). Leaf senescence dictates the duration of the functional source; delayed senescence maintains photosynthetic capacity but may impede nutrient remobilization to the sink (Luo et al., 2018; Dosio et al., 2024). Conversely, premature senescence induced by crowding or N deficiency limits the grain-filling period. An uncoordinated source-sink relationship often triggers premature senescence, yet the physiological interplay between leaf senescence timing and source-sink balance under varying N and density regimes requires further investigation. Understanding these physiological feedback loops is crucial for resolving the mechanisms of yield formation under stress.

While previous studies have established the agronomic benefits of density and N management, few have dissected the underlying physiological mechanisms regarding the interplay between source-sink relations and leaf senescence. Therefore, this study was conducted to: (1) quantify the dynamic changes in source characteristics and sink capacity, examining the yield-limiting physiological mechanisms through the lens of source-sink equilibrium; (2) analyze the synergistic effects of planting density and N rates on the regulation of leaf senescence and its subsequent feedback on source-sink relations; and (3) determine the physiological basis for maximizing water productivity and grain yield through optimized source-sink coordination. This study contributes to a mechanistic understanding of maize adaptation to arid environments.

## 2 Materials and methods

### 2.1 Experimental site

A two-year field experiment was conducted at the Agriculture and Ecological Water Conservation Experimental Station of China Agricultural University in Wuwei, Gansu Province, China (37° 52′ N, 102° 50′ E; 1580 m a.s.l.) from March to September in 2020 and 2021. This region experiences a temperate continental climate, with annual sunshine hours exceeding 3000 hours. Soil samples collected from the 0–40 cm layer exhibited the following physical properties: dry bulk density of 1.52 g cm-³, organic matter of 8.9 g kg-¹, total nitrogen of 0.5 g kg-¹, available phosphorus of 3.82 mg kg-¹, available potassium of 114.5 mg kg-¹, mineral nitrogen of 12.93 mg kg-¹, pH of 8.22, and field capacity of 32.9%. A standard meteorological station (Hobo, Onset Computer, USA) was utilized to collect meteorological data for the experiment. The reference evapotranspiration (ET_0_) and precipitation during maize growth periods are shown in **Supplementary Figure S1**.

### 2.2 Experimental design and field management

The widely cultivated maize variety “Zhengdan 958” was selected for this experiment. Maize seeds were sown on 8 May and harvested on 26 September in both years. The field experiment was arranged as a split‐plot design with three replicates. Planting density was assigned to the main plots and nitrogen rate to the subplots within each main plot. Each subplot measured 4 m in length and 5 m in width. The experimental treatments consisted of three planting densities (LD: 80,000 plants ha-¹, local planting density; MD: 100,000 plants ha-¹; HD: 120,000 plants ha-¹) and four nitrogen rates (N0: 0 kg N ha-¹; N1: 120 kg N ha-¹; N2: 180 kg N ha-¹; N3: 240 kg N ha-¹, local nitrogen rate). All treatments were planted in alternating wide and narrow rows (40–80 cm), with within-row plant spacing adjusted according to the target planting density. Maize growth periods in 2020 and 2021 are shown in **Supplementary Figure S2**. Other field management practices followed local practices. The fertilizers used in the region included urea (N-46%), potassium sulfate (K-52%), and calcium superphosphate (P-46%). Calcium superphosphate and potassium sulfate were applied at a rate of 100 kg per hectare across all treatments. Fertilization was conducted at the seedling, jointing, tasseling, and grain-filling stages of maize in the proportions of 20%, 30%, 30%, and 20%, respectively. Maize was irrigated every ten days, with dates adjusted based on weather conditions. The irrigation amount was determined by calculating 80% of the total crop water requirement (ETc) over the past 10 days, with seasonal irrigation amounts recorded as 317 mm in 2020 and 292 mm in 2021.

### 2.3 Plant sampling

#### 2.3.1 Leaf area index and aboveground dry matter

The leaf area index (LAI) and aboveground dry matter were assessed on six occasions throughout the six critical reproductive periods of maize. Within each plot, three maize plants were randomly selected to ascertain LAI and dry matter. LAI calculations followed the methodology of (Chan et al., 1986). For dry matter analysis, samples were first heated to 105 °C for 30 minutes to inactivate them, followed by drying at 80 °C until stable weight was achieved.

#### 2.3.2 Leaf area senescence dynamics

At the onset of the silking stage, 10 representative maize plants were marked in each plot. The green area of the ear leaves of the marked plants was measured every seven days from the silking stage to maturity, using the following formula (Chan et al., 1986):


GLA=0.75*L*W

where L and W denote the greatest length and maximum width of the leaf’s green portion, respectively.

The dynamics of green leaf area (GLAear) of spike leaves were fitted using a logistic equation:


$$y=\frac{a}{1+e^{-b\left(t-c\right)}}$$

where t represents the days post-silking (d), y denotes GLAear (cm²), a signifies the theoretical initial value of GLAear, b is a parameter characterizing the rate of leaf functional decline, and c indicates the time at which the maximum rate of decline occurs. According to Van Oosterom et al. (1996), the two points of inflection (t1, t2) and the corresponding GLAear (Y1, Y2) of the green leaf area dynamics could be calculated, with the calculations of other parameters carried out based on the calculated t1 and t2 as follows:


$$t_{1}=\frac{bc-\ln\left(2-\sqrt{3}\right)}{b},Y_{1}=\frac{a}{1+e^{-b\left(t_{1}-c\right)}}$$

$$t_{2}=\frac{bc-\ln\left(2+\sqrt{3}\right)}{b},Y_{2}=\frac{a}{1+e^{-b\left(t_{2}-c\right)}}$$

$$T_{1}=t_{1},V_{1}=\left(Y_{1}-a\right)/T_{1}$$

$$T_{2}=t_{2}-t_{2}$$

$$V_{2}=\left(Y_{2}-Y_{1}\right)/T_{2}$$

$$GLAD1_{ear}=\int_{0}^{t_{1}}\frac{a}{1+e^{-b\left(t-c\right)}}dt$$

$$GLAD2_{ear}=\int_{t_{1}}^{t_{2}}\frac{a}{1+e^{-b\left(t-c\right)}}dt$$

$$t_{0}=\frac{2.9444}{b}+c$$

$$T_{max}=c$$

$$V_{max}=\frac{ab}{4}$$

where T_1_ represents the duration of the initial phase of leaf functional decline, V_1_ the corresponding rate of decline, and GLAD_1ear_ the cumulative green leaf area during this phase. Similarly, T_2_ denotes the duration of the rapid functional decline phase, V_2_ the rapid decline rate, and GLAD_2ear_ the associated green leaf area duration. The green leaf area duration of spike leaves during the initial senescence phase GLAD_1ear_ was defined as the cumulative green leaf area from silking (t = 0) to the first inflection point (t = t_1_), the total area under the fitted logistic curve up to t_1_. This integral represents the cumulative GLA_ear_ at time t_1_. It was obtained analytically from the logistic function (closed-form solution) following the procedure described by Lan et al. (2024).GLAD2_ear_ was characterized as the area beneath the logistic curve from t_1_ to t_2_ post-anthesis (Van Oosterom et al., 1996). The variable t_0_ was defined as the time when GLA_ear_ declined to 95% of its theoretical initial value, marking the onset of detectable post-silking leaf functional decline. T_max_ represents the time required to reach the maximum rate of functional decline, and V_max_ denotes this maximum rate.

#### 2.3.3 Grain weight dynamics

In each plot, sixty maize plants were selected for analysis. Maize cobs were collected at five-day intervals throughout the grain-filling period. The harvested grains were dehooked, pooled, and randomly counted. The entire set of kernels was then placed in an oven at 105 °C for 30 minutes to inactivate them, followed by further drying at 75 °C until a stable weight was achieved (Thiex and Van Erem, 1999).

A sigmoid growth model was employed to describe the progression of overall maize kernel weight over time (Yin et al., 2009):


$$GW=\{\begin{array}{c} GW_{max}\times \left(1+\frac{t_{e}-t}{t_{e}-t_{m}}\right){\left(\frac{t}{t_{e}}\right)}^{\frac{t_{e}}{t_{e}-t_{m}}}\;\;\;\;\;\;\;\;if\;0\leq t\leq t_{e}\\ GW_{max}\;\;\;\;\;\;\;\;\;\;\;\;\;\;\;if\;t_{e}<t \end{array}$$

$$r_{a}=\frac{GW_{max}}{t_{e}}$$

$$r=r_{m}\left(\frac{t_{e}-t}{t_{e}-t_{m}}\right){\left(\frac{t}{t_{m}}\right)}^{\frac{t_{m}}{t_{e}-t_{m}}}$$

$$r_{m}=GW_{max}\left(\frac{2t_{e}-t_{m}}{t_{e}\times (t_{e}-t_{m})}\right){\left(\frac{t_{m}}{t_{e}}\right)}^{\frac{t_{m}}{t_{e}-t_{m}}}$$

$$RGR=\frac{(2t_{e}-t_{m})\left(t_{e}-t\right)}{(t_{e}-t_{m})\left(2t_{e}-t_{m}-t\right)t}$$

where GW is the grain weight (t); GW_max_ is the maximum grain weight when the grain filling stage lasts t_e_ (days); t represents the number of days following anthesis; tm indicates the point in time when the maximum rate of grain weight increase is attained; ra denotes the average rate of grain growth per day (t d^−1^); equation (4) is derived from equation (2); r_m_ signifies the maximum rate of grain growth per day (t d^−1^); and RGR refers to the relative rate of grain growth per day (t d^−1^ t^−1^).

#### 2.3.4 MDA content

MDA content was determined following the method of Heath and Packer (1968). MDA reacted with thiobarbituric acid (TBA) under high temperature and acidic conditions to form a colored trimethyl complex with light absorbance at 532 nm, exhibiting an absorbance coefficient of 155 [mmol (L^-1^ cm)^-1^] and a minimum absorbance at 600 nm. To eliminate sucrose interference, absorbance values at 450 nm, 532 nm, and 600 nm were measured. MDA content (C, nmol g^-¹^ FW) per unit weight of fresh tissue was calculated from the absorbance values at the three specified wavelengths.

#### 2.3.5 Source-sink relationships

##### 2.3.5.1 Source characteristics

The duration of leaf area at the canopy scale (LAD, expressed in square meters per day per square meter, m²·d m-²) was calculated using the following method (Li et al., 2022):

$$LAD_{pop}=\int LAI\left(t\right)dt$$

where LAI(t) represents the function of leaf area index fitted over time t.

The estimation of LAI was conducted as follows (Yin et al., 2003):


$$A=A_{\max}\left(1+\frac{t_{e}-t}{t_{e}-t_{m}}\right){\left(\frac{t}{t_{e}}\right)}^{\frac{t_{e}}{t_{e}-t_{m}}}$$

where A represents the leaf area index, t represents the number of days after sowing (d), A_max_ is the maximum value of LAI which is reached at t_e_ (as tasseling stage), and t_m_ is the time which reached the maximum linear growth rate.

##### 2.3.5.2 Source growth, sink capacity and source-sink activity

The sigmoid growth function was utilized to describe the dynamic growth pattern of maize dry matter, considering its varying properties (Yin et al., 2003; Yan et al., 2022):


$$DW=\{\begin{array}{c} DW_{max}\times \left(1+\frac{T_{e}-T}{T_{e}-T_{m}}\right){\left(\frac{T}{T_{e}}\right)}^{\frac{T_{e}}{T_{e}-T_{m}}}\;\;\;\;\;\;\;\;if\;0\leq T\leq T_{e}\\ DW_{max}\;\;\;\;\;\;\;\;\;\;\;\;\;\;\;\;\;\;\;\;\;\;\;if\;T_{e}<T \end{array}$$

$R_{a}=\frac{DW_{max}}{T_{e}}$


$$R=R_{m}\left(\frac{T_{e}-T}{T_{e}-T_{m}}\right){\left(\frac{T}{T_{m}}\right)}^{\frac{T_{e}}{T_{e}-T_{m}}}$$

$R_{m}=DW_{max}\left(\frac{2T_{e}-T_{m}}{T_{e}\times (T_{e}-T_{m})}\right){\left(\frac{T_{m}}{T_{e}}\right)}^{\frac{T_{m}}{T_{e}-T_{m}}}$


$$RGR=\frac{(2T_{e}-T_{m})\left(T_{e}-T\right)}{(T_{e}-T_{m})\left(2T_{e}-T_{m}-T\right)T}$$

In these equations, aboveground biomass after the days of silking is denoted as DW (kg ha-¹). T_e_ represents the time at which the maximum aboveground biomass is achieved (d). DW_max_ refers to the peak aboveground biomass observed during the reproductive phase. Total source growth (SG) is denoted by DW_max_. The peak grain weight signifies sink capacity (SC). The average aboveground biomass accumulation growth rate is designated as R_a_ (kg ha^-¹^ d^-¹^), while R (kg ha^-¹^ d^-¹^) signifies the instantaneous aboveground biomass growth rate at time t. Tm represents the time (d) at which the peak dry matter growth rate was recorded, while Rm signifies the maximum dry matter growth rate, which can be associated with either source activity (SCA), referring to aboveground biomass (kg ha^-¹^ d^-¹^), or sink activity (SIA), referring to grain dry weight. Lastly, RCR stands for the relative rate of dry matter growth.

The calculation of the disparity between sources and sinks, termed as DSS, was performed using the following formula:


DSS=SG−SC

The source-supply rate (SUR, kg ha-¹ d-¹) and sink growth rate (SGR, kg ha-¹ d-¹) were determined using the following equations based on previous studies (Shao et al., 2021; Li et al., 2022). SUR is indicative of source activity, while the exchange rate represents the sink’s demand during the grain-filling stage. The calculations for SUR and SGR are detailed below:


$$SUR=\frac{TB_{2}-TB_{1}}{t_{2}-t_{1}}$$

$$SGR=\frac{Y_{2}-Y_{1}}{t_{2}-t_{1}}$$

where Y_2_ and Y_1_ denote the grain weights at times t_2_, which corresponds to maturity, and t_1_, which is the anthesis stage, respectively. Similarly, TB_2_ and TB_1_ represent the total biomass measurements at the maturity stage (t_2_) and the silking stage (t_1_).

#### 2.3.6 Grain yield and its components, water productivity, irrigation water productivity, nitrogen partial factor productivity and nitrogen agronomic efficiency

At the harvest stage, 20 ears of maize were randomly selected from four undisturbed rows located at the center of each plot for harvesting and evaluation. And the 20 ears analyzed for yield components were subsampled from the same plots used for total yield measurement. After drying to 14% moisture content, the test plots were threshed, and the grain weight was measured to calculate the final yield. Water productivity (WP) and irrigation water productivity (IWP) were calculated using the following formulas:


$$WP=\frac{GY}{ET}$$

$$IWP=\frac{GY}{I}$$

where ET represents crop evapotranspiration (mm), and I represent the total irrigation water applied during the growing season (mm).

Crop evapotranspiration (ET) was calculated using the water balance approach, expressed as:


ET=P+U+I−R−D−ΔSWS

In this equation, P denotes precipitation (mm), U represents groundwater recharge (mm), I signifies irrigation volume (mm), R is surface runoff (mm), D refers to deep seepage (mm), and ΔSWS indicates the change in soil water storage (0–100 cm) between pre-sowing and post-harvest periods (mm). The experimental field was level, and its perimeter was surrounded by ridges to prevent runoff loss. Situated in an area with a groundwater depth exceeding 50 m, no groundwater recharge occurred. Moreover, over-irrigation was avoided, keeping the soil wetting depth below 2 m. As a result, U, R, and D were deemed negligible. Thus, Equation (31) was simplified to:


ET=P+I−ΔSWS

Soil water content (SWC, gravimetric method, hereafter) was determined at the seedling stage (V3) and physiological maturity (R6) in both 2020 and 2021 using the oven-drying method. At each stage, soil samples were collected from the 0–100 cm profile of each plot at 20 cm intervals with a soil auger (20 cm in length, 50 mm in diameter). Three sampling positions were selected in each plot: directly beneath the drip line, beneath the maize plant, and midway between the two. Soil water content was measured by drying the samples at 105 °C for 24 h. Soil water storage (SWS, mm) was then calculated as follows:


$$SWS=\sum_{i=1}^{n}swc_{i}\times \rho_{i}\times h_{i}\times 10$$

Where SWC*_i_* is the gravimetric soil water content (%) in layer *i*, ρ*_i_* is the bulk density (g cm^−3^), and h*_i_* is the thickness of the soil layer (cm). The indices *i* = 1,2,3,4,5 correspond to the 0–20, 20–40, 40–60, 60–80, and 80–100 cm soil layers, respectively.

Nitrogen partial factor productivity (NPFP) was calculated using the following formula:


$$NPFP=\frac{GY}{Amount\;of\;N\;applied}$$

Where GY is the grain yield (kg ha-1).


$$NAE=\frac{\left(GY_{N}-GY_{0}\right)}{Amount\;of\;N\;applied}$$

Where GY_N_ is the yield in the fertilized plot, GY_0_ is the yield in the unfertilized plot.

### 2.4 Statistical analysis

One-way ANOVA was performed using IBM SPSS Statistics 26 software (IBM Corp, Armonk, NY, USA) to test the significance of single-factor effects on the dependent variables, as well as to assess interactions between factors. *Post-hoc* multiple comparisons were conducted to evaluate differences among groups. Regression equations were constructed and illustrated using Origin 2024 software (Origin Lab Corporation, Northampton, MA, USA). The partial least squares structural equation modeling (PLS-SEM) was implemented in Smart PLS 4.0 software to investigate the interrelationships among different functional traits. The PLS-SEM was chosen for its minimal requirements regarding measurement scales, sample size, and residual distribution, making it well-suited for revealing the inherent characteristics of observed data. In this study, the goodness-of-fit of the PLS-SEM model was evaluated using the coefficient of determination (R²), Stone-Geisser coefficient (Q²), and the overall model fit (GoF) index (Wang et al., 2023). R² values above 0.19, 0.33, and 0.67 were categorized as weak, moderate, and strong, respectively. Q² values greater than zero indicated acceptable predictive relevance of the model for endogenous latent variables. GoF values above 0.1, 0.25, and 0.36 were classified as weak, moderate, and strong, respectively. The linear regression analysis was performed on the observed data to assess the relationships between functional traits. Regression lines were plotted in all relevant figures, with linear relationships achieving significance at the p< 0.05 threshold. Graphical illustrations were created using Origin 2024 (OriginLab Corp., Northampton, MA, USA).

## 3 Results

### 3.1 Dynamics of leaf green area attenuation after silking

**Figure 1:**
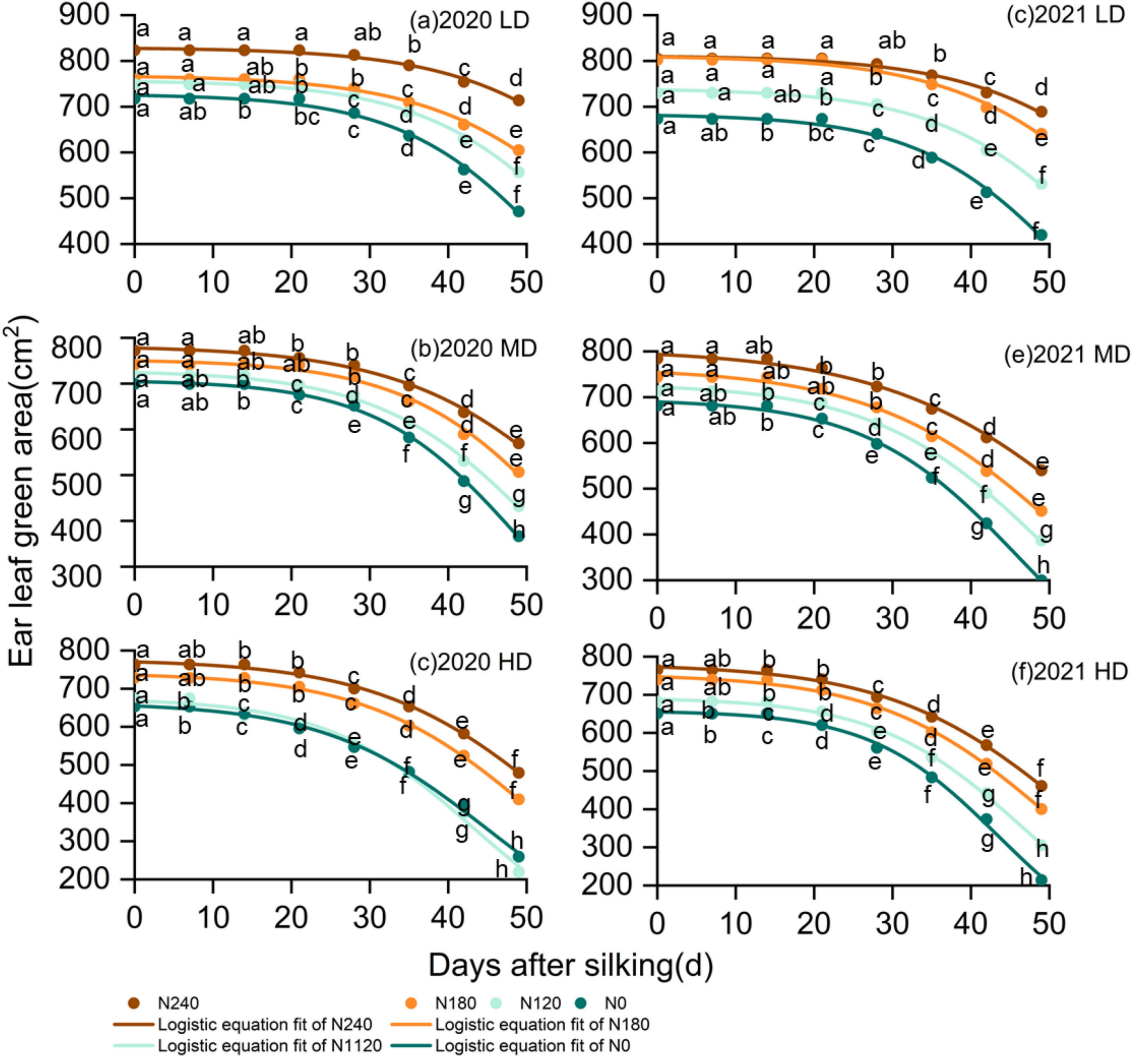
Dynamic changes of leaf green area (GLA_ear_) after silking. Different lowercase letters indicate significant differences among N rates at the same sampling time (p< 0.05).

As shown in **Figure 1**, the dynamics of GLA_ear_ fitted by the Logistic equation revealed that GLA_ear_ at DAS_14_ to DAS_21_ exhibited an initially slow decline that accelerated over time. The t_0_ (the time when GLA_ear_ diminished to 95% of the theoretical initial value) varied among different treatments. There were no significant differences in t_0_ among nitrogen rates, planting density, and their interaction (p >0.05) (**Tables 1**, **Supplementary Table S2**). Both planting density and nitrogen rate significantly affected the dynamics of ear-leaf green area attenuation after silking (**Tables 1**, **Supplementary Table S2**). Increasing nitrogen rate augmented “b,” and “c” exhibited a similar pattern to “b”, while higher planting density markedly diminished it. In contrast, higher planting density significantly reduced both parameters, indicating a more rapid decline in ear-leaf green area under dense planting conditions. With increasing planting density, T_1_ and GLAD_1ear_ decreased significantly. No significant difference in T_2_ was observed between the LD and MD treatments, whereas both were significantly higher 17.5% than that under the HD treatment (**Tables 1**, **Supplementary Table S2**). Neither V_max_ nor GLAD_2ear_ was significantly affected by planting density (p > 0.05). Nitrogen supply strongly influenced the timing and rate of ear-leaf green area attenuation. Compared with N0, the occurrence of rapid green area loss (T_1_) occurred significantly earlier under low nitrogen supply, whereas optimized nitrogen application (N1–N3) effectively alleviated the premature acceleration of green area attenuation, with T_1_ occurring 0.76, 1.9, and 3.9 days later under N1, N2, and N3, respectively. A similar pattern was observed for T_2_, which was delayed by 1.9, 5.2, and 7.0 days under N1, N2, and N3 relative to N0. note that GLAD_1ear_ and GLAD_2ear_ increased by 8.4%, 17.8%, and 21.4% under N1, N2, and N3, respectively, reflecting a sustained maintenance of ear-leaf green area after silking rather than a postponement of age-induced senescence. The parameter V_max_ gradually declined with increasing nitrogen rate, but remained significantly higher under LD and MD than under HD. No significant difference in V_max_ was observed between the N2 and N3 treatments at the same planting density.

**Table 1:** The logistic equation and its parameters for the dynamic change of ear leaf green area after silking in 2020.

2020	a	B	c		R^2^	t_0_	T_1_	V_1_	GLAD_1_ear	T_2_	V_2_	GLAD_2_ear	V_max_
LDN0	727.62b	-0.101bc		54.74c	0.997	25.59b	41.7d	-3.69b	28660.16d	26.08c	-16.11b	9486.63c	-18.37b
LDN120	757.65b	-0.095b		59.49b	0.998	28.5ab	45.63c	-3.51b	32675.87c	27.73b	-15.78ab	10538.91b	-17.99a
LDN180	768.46ab	-0.092ab		63.44ab	0.997	31.44a	49.13a	-3.31ab	35764.73b	28.63ab	-15.5a	11036.7ab	-17.67a
LDN240	829.16a	-0.084a		69.36a	0.999	34.31a	53.68a	-3.26a	42214.96a	31.36a	-15.27a	12976.35a	-17.41a
**AV.**	**770.7225A**	**-0.0925A**		**61.76A**		**29.96a**	**47.54a**	**-3.4425A**	**34828.93A**	**28.45A**	**-15.665A**	**11009.65A**	**-17.86A**
MDN0	707.7b	-0.108bc		49.52c	0.985	22.26b	37.33b	-4.01b	24898.88d	24.39c	-16.75c	8618.29c	-19.11c
MDN120	728.63b	-0.099b		52.66b	0.992	22.92ab	39.36ab	-3.91b	26999.9c	26.61b	-15.81b	9654.35b	-18.03b
MDN180	752.79ab	-0.094ab		55.94ab	0.995	24.62a	41.93ab	-3.79a	29746.35b	28.02ab	-15.51ab	10493.89ab	-17.69ab
MDN240	782.94a	-0.087a		60.53a	0.994	26.69a	45.39a	-3.64a	33435.62a	30.27a	-14.93a	11871.02a	-17.03a
**AV.**	**743.015AB**	**-0.0975A**		**54.66AB**		**24.12b**	**41b**	**-3.8375A**	**28770.19B**	**27.32AB**	**-15.75A**	**10159.39AB**	**-17.965A**
HDN0	663.41c	-0.115c		43.65b	0.968	18.05a	32.2b	-4.35bc	20035.25d	22.9c	-16.72bc	7589.41d	-19.07d
HDN120	674.91c	-0.109c		43.37b	0.969	16.36a	31.29b	-4.56c	19686.26c	24.16b	-16.13b	8173.16c	-18.39c
HDN180	741.59b	-0.093b		51.26a	0.985	19.6a	37.1a	-4.22b	25675.63ab	28.32a	-15.12a	10517.31ab	-17.24b
HDN240	777.02a	-0.087a		54.47a	0.988	20.63a	39.33a	-4.17a	28499.73a	30.27a	-14.82a	11787.39a	-16.9a
**AV.**	**714.2325B**	**-0.1025B**		**48.18B**		**18.66c**	**34.98c**	**-4.325B**	**23474.22C**	**26.41B**	**-15.6975A**	**9516.82B**	**-17.9A**
F-value													
D	ns	*		**		ns	**	**	**	**	ns	**	ns
N	**	**		**		ns	**	**	**	**	**	**	**
D×N	ns	ns		ns		ns	ns	ns	ns	ns	ns	ns	ns

a signifies the theoretical initial value of GLA_ear_, b is a parameter characterizing the rate of leaf functional decline, and c indicates the time at which the maximum rate of decline occurs. t_0_ is the time when the GLA_ear_ decreased to 95% of the theoretical initial value (d), T_1_ and T_2_ are the duration of the initial phase of leaf functional decline, V_1_ the corresponding rate of decline, and GLAD1_ear_ the cumulative green leaf area during this phase. Similarly, T_2_ denotes the duration of the rapid functional decline phase, V_2_ the rapid decline rate, and GLAD2_ear_ the associated green leaf area duration. V_max_ denotes this maximum rate of leaf functional decline. Different lowercase letters to the right of the value indicate differences among treatments at the same density (p< 0.05). Different uppercase letters to the right of the value indicate differences among density treatments (p< 0.05). *, p<0.05; **, p<0.01. ns, not significant.

The bold values represent the mean of each parameter under the same planting density, and the capital letters that follow indicate the significance of each parameter among different planting densities.

### 3.2 Changes in leaf membrane lipid peroxidation product malondialdehyde after silking

**Figure 2:**
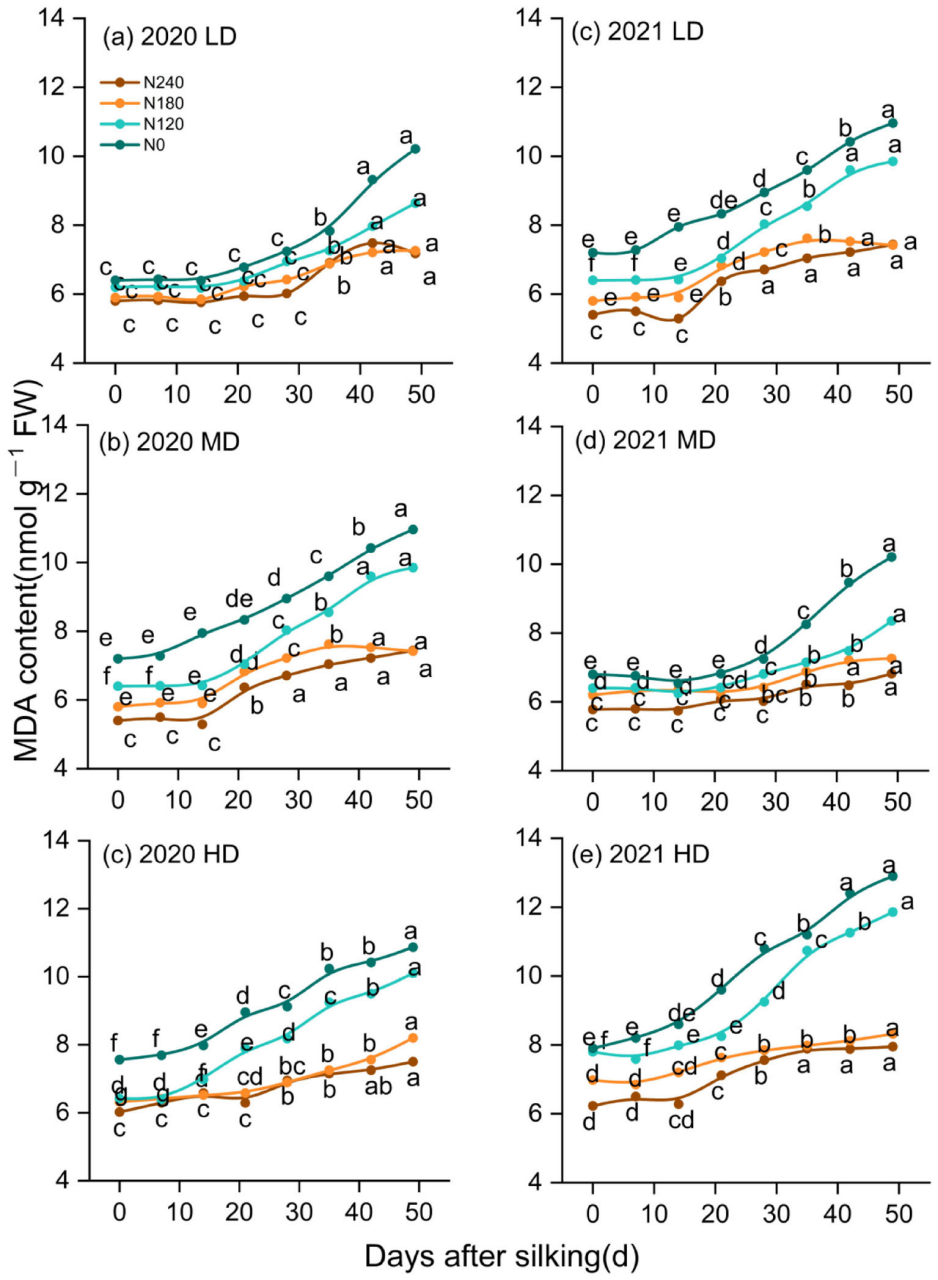
Dynamics of MDA content of leaf after silking. Different lowercase letters to the right of the value indicate differences among treatments at the same density at the same sampling time (p< 0.05).

The MDA content varied in response to different nitrogen rates and planting densities (**Figure 2**). Following silking, MDA levels remained relatively stable during the early period and subsequently increased as plants progressed toward later developmental stages. Low nitrogen supply accelerated the accumulation of MDA after silking, whereas optimized nitrogen application effectively mitigated oxidative damage associated with premature leaf functional decline. Compared to that of N0, the time t_o_ significant MDA increase was delayed by 16.3 days in N1, 9.3 days in N2, and 4.7 days in N3, respectively. There were no significant differences in the timing of MDA increases between LD and MD treatments, both of which experiencing an 8.75-day delay compared to the HD treatment. Compared to N0, MDA levels decreased by 5.6%, 35.0%, and 51.8% in N1, N2, and N3, respectively. In contrast, maximum MDA content increased significantly with planting density, showing increases of 9.5% and 17.4% under MD and HD, respectively, compared with LD. These results demonstrate that low nitrogen availability and high planting density accelerated oxidative stress development after silking, whereas optimized nitrogen management alleviated stress-induced premature leaf functional deterioration.

### 3.3 Leaf area index dynamics and source size

**Figure 3:**
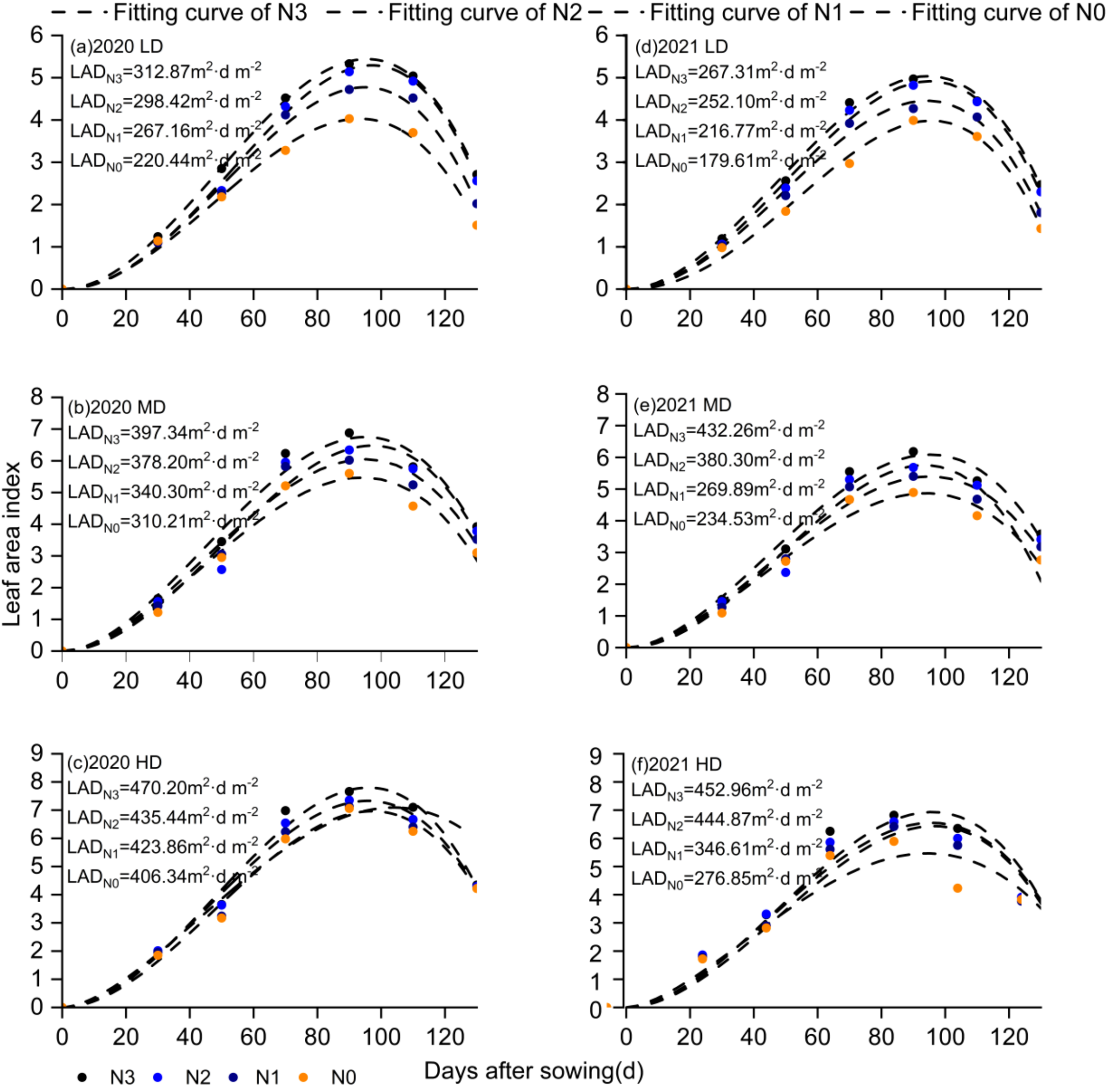
Measured and fitted (by the modified Logistic model) LAI and LAD among different treatments during the 2020 and 2021 maize growing seasons. The error bars indicate the standard error of the means (n = 3) at p< 0.05 level.

Leaves act as sites for converting solar energy into chemical energy, which provide energy for plant growth. The dynamics of leaf area under different planting densities and nitrogen rates exhibited “S” trends (**Figure 3**). Source strength (the size of the source and its activity) is generally characterized by leaf area duration (LAD), which significantly affects sink strength. The Logistic model was applied to fit the LAI of maize, with coefficients of determination ranging from 0.929 to 0.999, indicating an excellent fit. Compared to that of N0, LAD significantly increased by 13.9%, 34.4%, and 48.9% under N1, N2, and N3, respectively (**Figure 3**). Compared to that of LD, LAD significantly increased by 36.2% and 62.6% in MD and HD, respectively (**Figure 3**).

### 3.4 Source growth

**Figure 4:**
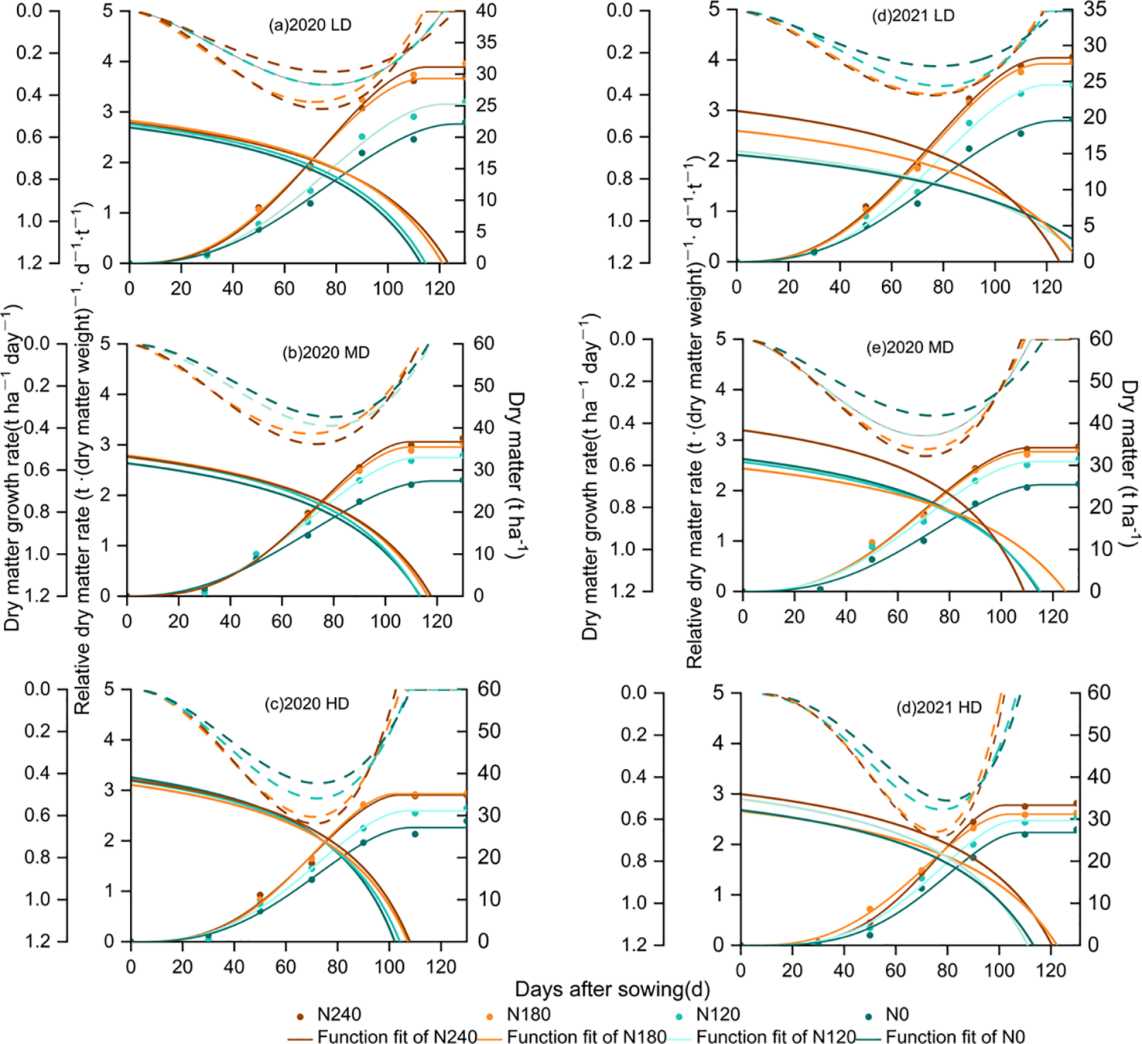
Measured and fitted dry matter in different treatments (lines with points) during the maize growing seasons in 2020 and 2021. Fitted dry matter growth rate (lines) in different treatments during the maize growing seasons in 2020 and 2021. Fitted relative dry matter growth rate (dashed lines) in different treatments during the maize growing seasons in 2020 and 2021.

The dry matter in various treatments initially grew, then experienced a rapid increase, followed by a gradual increase, ultimately stabilizing. The dry matter accumulation rate initially rose and then gradually diminished (**Figure 4**), while the relative growth rate showed a slow decline (**Figure 4**). Compared to that of N0, DM_max_ increased by 17.2% in N1, 29.5% in N2, and 31.7% in N3, respectively (**Table 2**). Compared to that of N0, T_e_ significantly prolonged by 9.1d in N3, 10.2 d in N2, and 6.5 d in N1, respectively; T_m_ advanced by 6.8 d in N3, 6.2 d in N2, and 2.5 d in N1, respectively. DM_max_ initially increased, then slightly decreased with increasing planting density, with DM_max_ under MD and HD showing substantial increases of 19.4% and 23.0% compared to LD, respectively. Planting density significantly advanced the appearance of T_e_ by 20.5 d under both MD and HD.

**Table 2:** Results of growth equations in simulating maize grain weight during the growing seasons of 2020 and 2021.

Year	Model parameters								Characteristic parameters			
Treatments	GW_max_(kg ha^1^)		t_e_(d)		t_m_(d)		R^2^		r_a_(kg ha^1^ d^-1^)		r_m_(kg ha^1^ d^-1^)	
	2020	2021	2020	2021	2020	2021	2020	2021	2020	2021	2020	2021
LDN0	9658	9000	42.44	44.75	22.2	21.65	0.996	0.993	227.568	201.117	346.223	299.12
LDN1	11386	10739	44.24	45.41	21.45	20.52	0.998	0.999	257.369	236.49	382.982	347.006
LDN2	14425	12975	45.55	47.86	21.6	19.83	0.993	0.994	316.685	271.103	468.885	393.539
LDN3	12962	13452	45.94	47.4	17.99	18.63	0.994	0.998	282.151	283.797	407.959	410.42
MDN0	10768	12087	43.39	45.66	22.06	21.89	0.998	0.996	248.168	264.717	374.071	392.883
MDN1	13555	14715	43.66	44.96	22.79	21.9	0.999	0.996	310.467	327.291	471.999	487.577
MDN2	15886	17873	44.57	46.24	22.47	22.84	0.999	0.998	356.428	386.527	535.909	577.872
MDN3	16279	17625	44.84	45.69	22.26	22.97	0.997	0.999	363.046	385.752	543.496	579.526
HDN0	12456	11363	39.64	39.94	26.91	26.41	0.997	0.998	314.228	284.502	570.031	501.474
HDN1	14121	12878	39.99	40.16	26.48	26.43	0.992	0.999	353.113	320.667	623.307	562.509
HDN2	15535	13336	40.33	40.31	25.13	25.26	0.996	0.991	385.197	330.836	643.758	555.38
HDN3	16213	14660	40.88	40.62	25.22	24.93	0.999	0.993	396.6	360.906	657.801	596.32

GW_max_ is the fitted maximum population grain weight, t_e_ is the duration of grain filling, t_m_ is the time when maximum grain filling rate occurs, r_a_ is the average grain filling rate, r_m_ is the maximum grain filling rate.

### 3.5 Grain weight growth process

**Figure 5:**
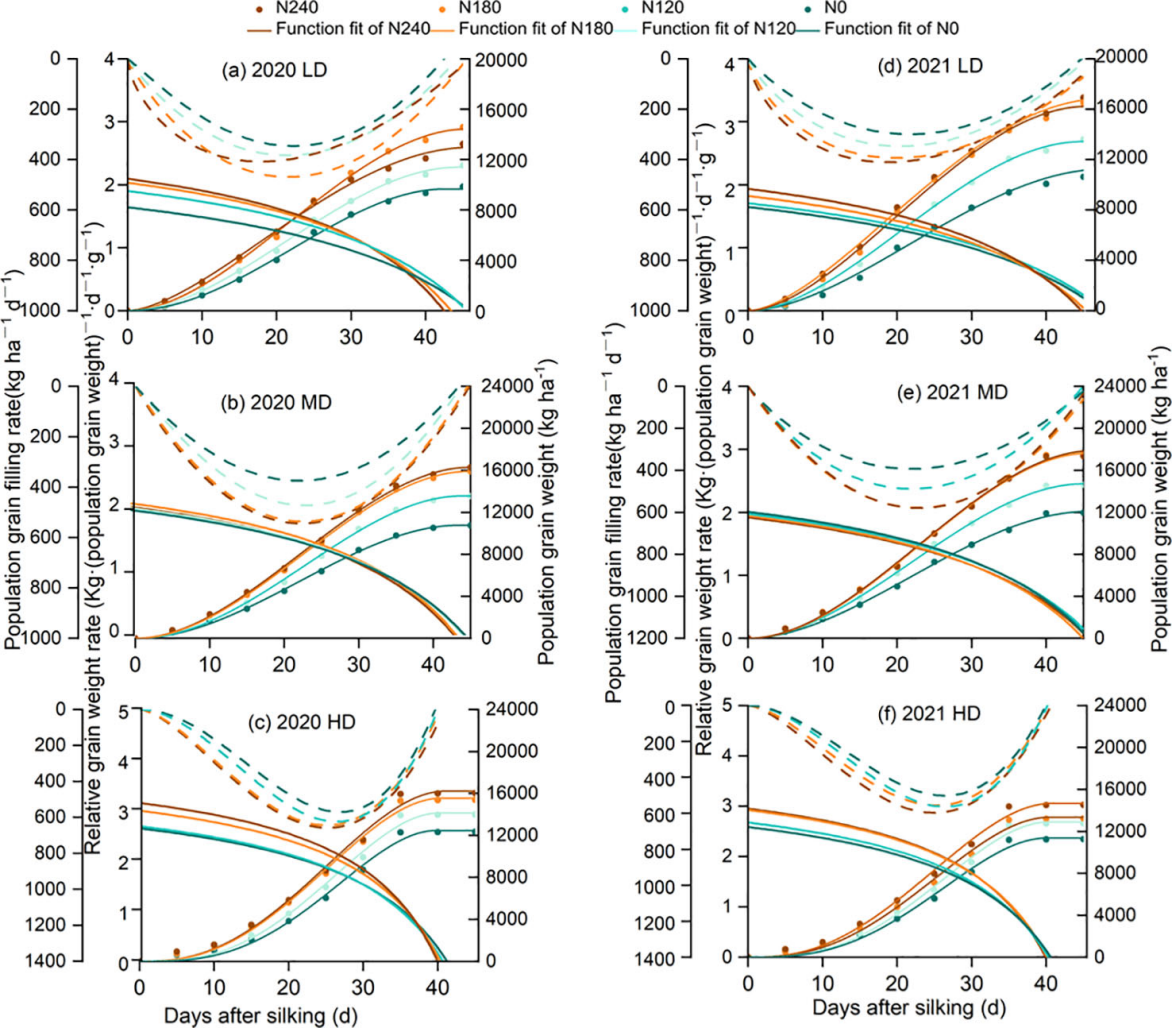
Simulated population grain weight among different treatments (lines with points) during the 2020 and 2021 maize growing seasons. The error bars indicate the standard error of the means (n = 3) at p< 0.05 level. Population grain filling rate (lines) in different treatments during the maize growing seasons in 2020 and 2021. The relative grain weight growth rate (dashed lines) in different treatments during the maize growing seasons in 2020 and 2021.

Growth rates in various treatments first increased before progressively declining, while the relative growth rate exhibited a steady decrease (**Figure 5**). Nitrogen application increased GW_max_ by an average of 21.5%, 44.1%, and 46.2% in N1, N2, and N3 compared to N0, respectively (**Table 3**). Additionally, nitrogen application delayed t_e_ by an average of 0.4 d, 1.5 d, and 1.6 d in N1, N2, and N3 relative to N0, while t_m_ was advanced by an average of 0.3 d, 0.6 d, and 1.5 d in N1, N2, and N3 compared to N0. GW_max_ initially increased and then decreased with planting density, achieving its maximum value at MD. The maximum value for GW_max_ increased significantly by an average of 16.9% and 25.6% in MD and HD compared to LD, respectively. Increasing planting density notably advanced the emergence time of t_e_, with MD and HD advancing by 0.6 d and 5.2 d compared to LD, respectively.

### 3.6 Source-sink relations

**Figure 6:**
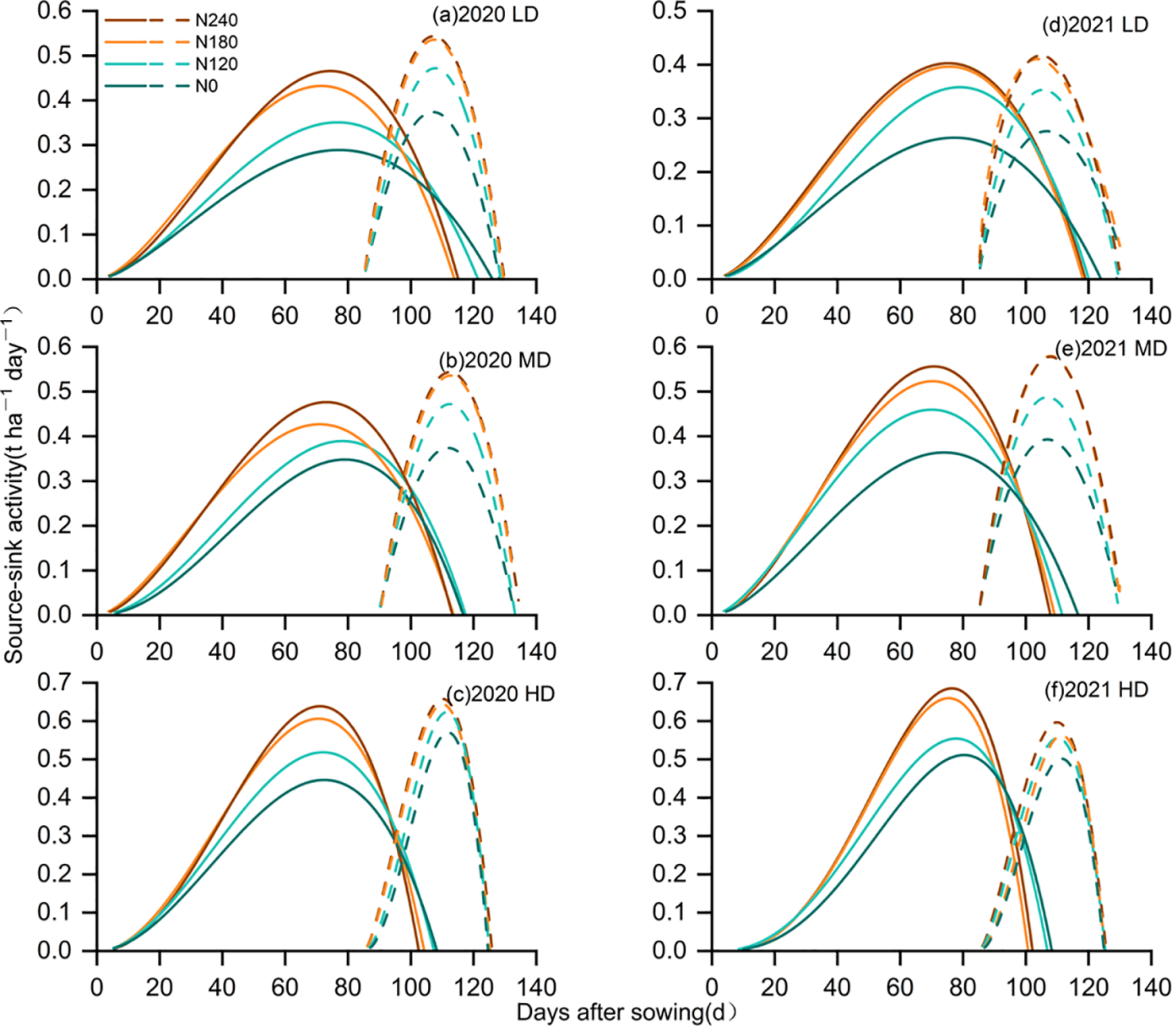
**(a)** Source-sink activity during the 2020 and 2021 growing seasons of maize in different. **(b)** The solid lines represent the source activity, and the dashed lines represent the sink activity.

The source activity peaked between 70 and 80 days, while sink activity peaked around 110 days, with the grain-filling period occurring approximately between 85 and 130 days. After the initiation of grain filling, a marked reduction in source activity was observed (**Figure 6**). Nitrogen application significantly improved the grain-filling rate (**Table 3**). Moreover, it increased the maximum source-sink growth value, resulting in an earlier peak. The termination of source growth occurred marginally before the end of sink growth. During the grain-filling phase, variations in source capacity were more significant than those in sink strength.

**Table 3:** Results of growth equations in simulating maize dry matter during the growing seasons of 2020 and 2021.

Year	Model parameters								Characteristic parameters			
Treatments	DM_max_ (t ha^-1^)		T_e_ (d)		T_m_ (d)		R^2^		R_a_ (t ha^-1^ d^-1^)		R_m_ (t ha^-1^ d^-1^)	
	2020	2021	2020	2021	2020	2021	2020	2021	2020	2021	2020	2021
LDN0	22.38	19.78	123.36	123.89	78.98	83.15	0.996	0.993	0.182	0.158	0.312	0.283
LDN1	25.61	24.74	121.15	119.58	78.33	81.59	0.998	0.999	0.213	0.203	0.367	0.37
LDN2	31.65	28.66	114.64	119.72	73	74.43	0.993	0.994	0.273	0.233	0.465	0.389
LDN3	29.41	29.43	112.82	117.89	70.92	73.65	0.994	0.998	0.257	0.23	0.432	0.385
MDN0	27.55	25.59	117.78	117.51	75.21	80.3	0.998	0.996	0.297	0.216	0.507	0.368
MDN1	33.47	31.51	116.46	111.26	74.69	76.41	0.999	0.996	0.301	0.278	0.514	0.49
MDN2	36.34	33.82	113.21	109.42	72.22	72.75	0.999	0.998	0.32	0.304	0.545	0.52
MDN3	37.54	34.5	113.47	107.48	70.4	71.85	0.997	0.999	0.33	0.318	0.55	0.56
HDN0	28.68	27.5	108.01	108.27	74.3	74.82	0.997	0.998	0.264	0.248	0.486	0.512
HDN1	31.77	30.36	107.23	106.44	72.74	73.66	0.992	0.999	0.295	0.258	0.534	0.504
HDN2	34.22	31.46	104.1	102.02	71.44	70.27	0.996	0.991	0.333	0.291	0.612	0.563
HDN3	34.86	33.77	102.15	100.46	70.79	70.92	0.999	0.993	0.343	0.311	0.637	0.604

DM_max_ is the max dry mass, T_e_ is the time when dry matter reaches its maximum, T_m_ is the time of maximum dry matter growth rate, R_m_ is the maximum dry matter growth rate, R_a_ is the average dry matter growth rate.

Source growth (SG), sink capacity (SC), and sink growth rate (SGR) gradually increased and then slightly decreased with increasing planting density (**Table 4**). Compared to those of LD, source growth (SG) increased by 23.0% in MD and 19.4% in HD, sink capacity (SC) increased by 23.9% in MD and 15.2% in HD, and sink growth rate (SGR) increased by 23.7% in MD and 15.8% in HD, respectively. The source-sink difference (DSS) gradually increased with increasing planting density. Compared to that of LD, DSS increased by 16.2% in MD and 18.2% in HD. Increasing planting density decreased the source-supply ratio (SUR) by 5.0% and 10.6% (SUR) in MD and HD compared to LD, respectively.

**Table 4:** Source growth (SG), sink capacity (SC), the difference value between source-sink (DSS), source supply rate (SUR) and sink growth rate (SGR) during the 2020 and 2021 maize growing seasons.

Year	SG		SC		DSS		SUR		SGR	
Treatments	2020	2021	2020	2021	2020	2021	2020	2021	2020	2021
LDN0	22.38	19.6	9847	8505	12.77	11.17	0.198	0.184	0.197	0.17
LDN1	25.61	24.27	11526	10885	14.49	14.08	0.219	0.24	0.231	0.218
LDN2	31.65	27.93	14560	13184	18.96	16.13	0.263	0.244	0.291	0.264
LDN3	29.41	27.12	13235	13538	18.02	16.05	0.207	0.24	0.265	0.271
MDN0	27.55	25.4	11977	10749	15.27	14.65	0.17	0.163	0.24	0.215
MDN1	33.47	30.91	14610	13551	18.82	18.03	0.218	0.244	0.292	0.271
MDN2	36.34	33.22	17644	15896	18.7	17.62	0.238	0.209	0.353	0.318
MDN3	37.54	34.2	17444	16217	20.09	18.18	0.247	0.213	0.349	0.324
HDN0	28.68	26.85	12333	11550	16.47	15.88	0.188	0.206	0.247	0.231
HDN1	31.77	27.43	13984	12753	18.09	17.65	0.197	0.207	0.28	0.255
HDN2	34.22	29.68	15400	13215	18.9	18.62	0.188	0.173	0.308	0.264
HDN3	34.86	31.22	16037	14520	19.06	19.11	0.215	0.209	0.321	0.29

Increasing nitrogen rate improved source-sink parameters (SG, SC, DSS, SUR, and SGR), but there were no significant differences in these indicators between N2 and N3. Compared to N0, SG improved by 17.2% in N1 and 29.5% in N2, SC improved by 19.0% in N1 and 38.4% in N2, DSS improved by 17.3% in N1 and 26.4% in N2, SUR improved by 19.8% in N1 and 18.0% in N2, and SGR improved by 19.2% in N1 and 37.7% in N2.

### 3.7 Grain yield components, WP, IWP, NPFP and NAE

**Figure 7:**
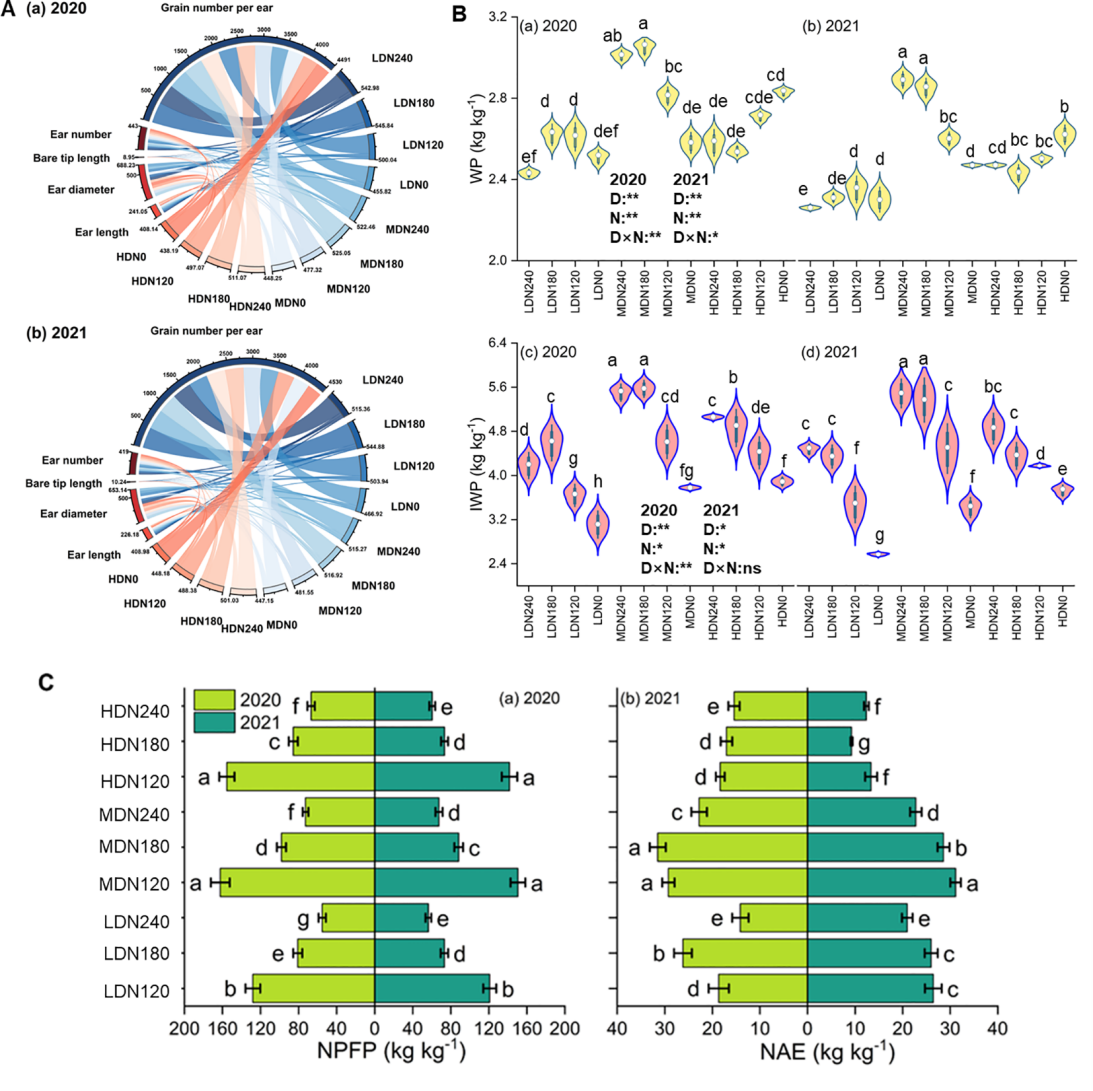
**(A)** chord diagram of yield components for each treatment in 2020 (a) and 2021 (b); **(B)** water productivity (WP) and irrigation water productivity (IWP); **(C)** nitrogen partial factor productivity (NPFP) and nitrogen agronomic efficiency (NAE) under different planting densities (D) and nitrogen application rates (N). Error bars represent the standard deviation (SD). Lowercase letters indicate statistically significant groups at p< 0.05.

Planting density, nitrogen rate, and their interaction have a significant impact on the yield components (p< 0.05) (**Figure 7A**). Increasing planting density showed a downward trend in the ear number, ear diameter, ear length, while the bare tip length increased. Nitrogen application significantly improved ear number, ear diameter and ear length, but the response to nitrogen supply varied significantly at different planting densities. At LD and MD, nitrogen application up to 180 kg ha^-1^ achieved the highest values for these indicators, which were significantly higher than those of non-nitrogen treatment. Further increases in nitrogen rate did not significantly enhance these indicators. At HD, these indicators reached their maximum values under N3, which were significantly higher than those of N2 and N0.

Water productivity (WP) increased with planting density, but the differences were not significant (**Figure 7B**). Compared to LD and HD, WP at MD increased significantly by 12.5% and 8.0%, respectively. At LD, WP first increased and then decreased with increasing nitrogen rate, reaching its peak at N2, which was 7.8% higher than that at N3. At MD and HD, WP increased with higher nitrogen rates. The changes in irrigation water productivity (IWP) were consistent with those in grain yield.

NPFP reached its maximum value at MDN1 in both years, and was significantly higher than all LD and HD treatments in the same year, showing a trend of N1 > N2 > N3 under the same planting density (**Figure 7C**). NAE was also highest under medium density and was significantly higher than that under HD. At the high N application level (N3), both NPFP and NAE were relatively low and were significantly reduced by 57% and 16%, respectively, compared with N1, indicating that excessive N application markedly decreases N use efficiency. In summary, both NPFP and NAE remained at relatively high levels under the MDN1 and MDN2 treatments.

### 3.8 Relationships among traits

**Figure 8:**
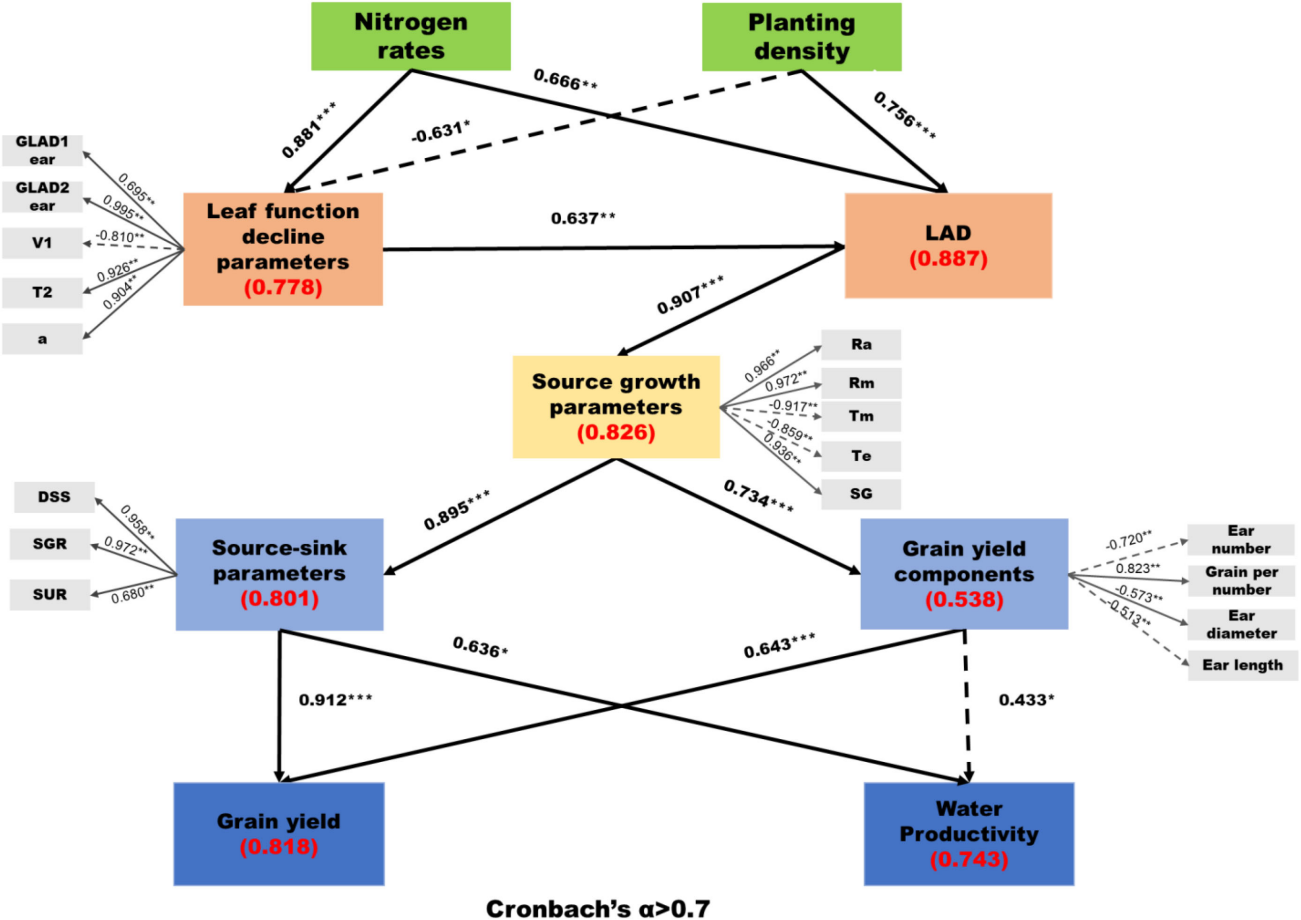
PLS-SEM evaluation of direct and indirect relationships among leaf function decline parameters, source and sink. The traits used in PLS-SEM include leaf function decline parameters (a: the theoretical initial value of GLA_ear_, V_1_: the corresponding rate of decline, GLAD1_ear_: the cumulative green leaf area during T_1_, T_2_: the duration of the rapid functional decline phase, GLAD2_ear_: the associated green leaf area duration, V_max_: this maximum rate of leaf functional decline), LAD, source growth parameters (R_a_: the average dry matter growth rate, R_m_: the maximum dry matter growth rate, T_m_: the time of maximum dry matter growth rate, T_e_: the time when dry matter reaches its maximum, SG: source growth), source-sink parameters (DSS: the difference value between source-sink, SGR: sink growth rate, SUR: source supply rate), grain yield components (ear length, ear diameter, ear number, grain per number) and grain yield (SC). The numbers next to the arrows indicate standardized path coefficients (β). Solid arrows and dashed arrows represent positive and negative relationships, respectively. Asterisks indicate significance (Bootstrapping analysis: *p< 0.05, **p< 0.01, ***p< 0.001), and “ns” indicates no significant difference. Adjusted coefficients of determination (R²) and Stone-Geisser coefficients (Q²) are displayed.

Partial least squares structural equation modeling (PLS-SEM) was employed to analyze multi-hypotheses in the path diagram, elucidate causal relationships among post-silking leaf functional decline source-sink relations, grain yield components, grain yield, and water productivity and evaluate the direct and indirect effects of planting density (D) and nitrogen rate (N) on these traits. The optimal model included six variables (leaf functional decline indicators, LAD, source growth parameters, source-sink parameters, grain yield components, grain yield, and water productivity) and core observed variables representing the latent variables (**Figure 8**). The PLS-SEM exhibited high reliability and validity, with a goodness-of-fit (GoF) value of 0.584. The average variance extracted (AVE) for all latent variables exceeded 0.5, and both Cronbach’s alpha and composite reliability (CR) values were greater than 0.7 (**Supplementary Table S1**). Nitrogen rate exerted a direct and significant positive effect on LAD (β = 0.666, p< 0.01) and a significant negative effect on indicators of premature leaf functional decline (β = −0.881, p< 0.01), indicating an alleviation of stress-induced early loss of leaf function. In contrast, planting density had a direct and significant positive effect on leaf functional decline indicators (β = 0.756, p< 0.01) and a significant negative effect on LAD (β = −0.631, p< 0.05), reflecting intensified competition-induced reductions in effective canopy function. Both nitrogen rates and planting density significantly influenced source growth parameters indirectly through their effects on leaf functional decline and LAD (β = 0.907, p< 0.001), but neither had a direct effect on source growth parameters (P > 0.05, not shown in the path diagram) (**Figure 8**). Additionally, source growth parameters directly influenced source-sink parameters and grain yield components (β = 0.895, p< 0.001; β = 0.734, p< 0.001; **Figure 8**). On the other hand, source-sink parameters and grain yield components had direct and significant positive effects on grain yield (β = 0.912, p< 0.001; β = 0.636, p< 0.001). Also, source-sink parameters had direct significant positive effects on water productivity (β = 0.636, p< 0.05), and grain yield components had direct significant negative effects on water productivity (β = 0.433, p< 0.05).

## 4 Discussion

### 4.1 Effects of planting density and nitrogen rate on leaf senescence and dry matter translocation after silking

**Figure 9:**
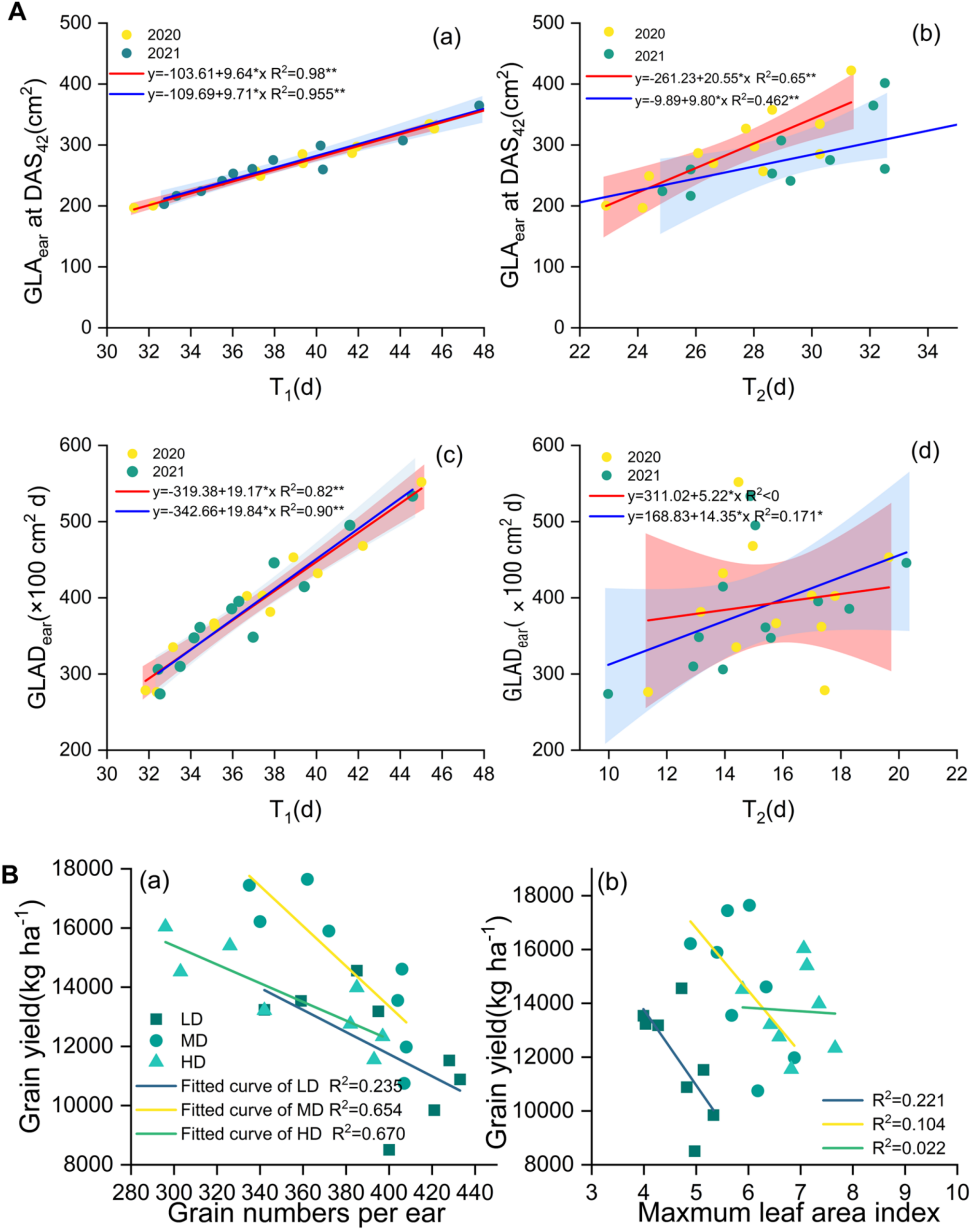
**(A)** relationships between T_1_ (a), T_2_ (b) and GLAear at DAS_42_ (c), relationships between T_1_ **_(_c_)_**, T_2_ (d) and GLAD_ear_ in 2020 and 2021. **(B)** relationships between grain numbers per ear (a), maximum leaf area index (b) and grain yield in different densities.

Increasing nitrogen supply significantly influenced post-silking leaf physiological dynamics, as reflected by reduced rates of leaf functional decline (V_1_, V_2_, and V_max_; **Tables 1**, **Supplementary Table S2**) and an extended duration of effective vegetative growth. Low nitrogen supply accelerated the progression of leaf functional deterioration, whereas optimized nitrogen application alleviated premature leaf senescence by sustaining chlorophyll content and maintaining leaf green area after silking. The parameters T_1_ and T_2_, which characterize the timing of rapid leaf functional decline, were closely associated with GLAD_1ear_ and GLAD_2ear_ (**Figure 9A**; Wang et al., 2023; Lan et al., 2024), indicating that nitrogen availability primarily affected the rate and onset of post-silking leaf deterioration rather than postponing age-induced senescence. The physiological basis for this response is likely related to nitrogen-mediated regulation of chlorophyll metabolism and oxidative stress. Adequate nitrogen supply has been shown to suppress the activity of chlorophyll-degrading enzymes, enhance antioxidant enzyme systems, and inhibit premature chlorophyll breakdown (Ouyang et al., 2018; Mu and Chen, 2021; Li et al., 2024). Consistent with this mechanism, our results demonstrated that increasing nitrogen rate significantly reduced both the accumulation and accumulation rate of malondialdehyde (MDA) in post-flowering leaves (**Figure 2**), indicating alleviated lipid peroxidation and improved membrane integrity. In contrast, under low nitrogen conditions, protein degradation during the senescence process was more severely affected by age-related oxidative damage to functional proteins (Havé et al., 2017).

High planting density intensified interplant competition and canopy shading, leading to a higher proportion of far-red light and inducing stress-related acceleration of leaf functional decline (Shao et al., 2021) In addition, competition for water, light, and nutrients under dense planting constrained nitrogen availability at the individual plant level, thereby limiting the maintenance of high nitrate reductase and carboxylase activities (Craine and Dybzinski, 2013; Lan et al., 2024; Wei et al., 2024). These constraints resulted in elevated MDA accumulation (**Figure 2**), reflecting enhanced oxidative damage to leaf cell membranes. By contrast, under low- and medium-density conditions, improved light interception and resource availability enhanced light-use efficiency (Lai et al., 2025), reduced excessive reactive oxygen species (ROS) production, and promoted the activities of antioxidant enzymes such as SOD and POD (Zhang et al., 2007), thereby mitigating lipid peroxidation.

It should be noted that although sufficient nitrogen supply helps maintain post-silking leaf function, excessive nitrogen input may disrupt the balance between photosynthetic maintenance and nutrient remobilization. An overly prolonged functional state of leaves may impair the timely translocation of nitrogen and assimilates from vegetative organs to developing grains (Gregersen et al., 2013; Liu et al., 2023), potentially constraining dry matter accumulation and transport after the milk stage (Joshi et al., 2019). Therefore, appropriate nitrogen management is essential to alleviate premature leaf senescence while ensuring timely nutrient remobilization, thereby achieving an optimal balance between sustained photosynthesis and efficient source–sink coordination under different planting densities.

### 4.2 Effects of planting density and nitrogen rate on source-sink relationships

#### 4.2.1 Dynamics of leaf area index and source growth

Source organs and their productivity form the foundation of yield development (Li et al., 2022). Leaf area and leaf area duration (LAD) are critical factors determining dry matter accumulation and increases in corn yield (Wang et al., 2017). Our study found that while increasing planting density significantly enhanced leaf area index, excessively high leaf area index was not a necessary condition for yield improvement. This was because leaf area duration exhibited a strong positive correlation with yield (Liu et al., 2017, Liu et al., 2023).

Nitrogen application significantly increased dry matter content and promoted source growth (DM_max_) (Ma et al., 2021; Mu and Chen, 2021). Under LDN3, DM_max_ showed a slight downward trend compared to LDN2 (**Table 2**). This may be due to that excessive nitrogen application significantly increased stem non-structural carbohydrate reserves, thereby reducing the amount transferred to grains (Liu et al., 2017; Liang et al., 2019). Under medium-density (MD) planting treatments, the slight decrease in DM_max_ under high-density (HD) treatments was primarily due to reduced available resources per plant (Chen et al., 2015), accelerated leaf senescence (Antonietta et al., 2014), and a decline in net photosynthesis per plant after silking (Tokatlidis et al., 2011). Additionally, our study indicated that increasing nitrogen rate and planting density advanced the timing of maximum dry matter growth rate and accumulation (t_m_ and T_m_), while also increasing average and maximum dry matter growth rates (R_a_ and R_m_). Between high-density (HD) and medium-density (MD) treatments, Ra showed no significant differences, but Rm was significantly higher under HD. This was because shading caused by high planting density accelerated the decline of post-silking leaf functional activity, thereby shortening the linear growth phase of maize (Wu et al., 2023).

#### 4.2.2 Dynamics of grain weight

Grain weight followed “S” growth patterns, exhibiting “slow-fast-slow” growth trends, consistent with previous studies (Jia et al., 2018; Cao et al., 2024). Increasing planting density accelerated grain weight growth, while t_max_ (the time when grain weight reaches its peak) was delayed with increasing planting density (**Table 3**). These factors were key reasons for reduced grain yield under high-density planting (Liu et al., 2017; Arata et al., 2023). Between low-density (LD) and medium-density (MD) treatments, t_e_ (the time when grain weight begins rapid growth) and t_m_ (the time when grain weight reaches its peak) showed no significant differences, as maize plants exhibited similar light interception rates under LD and MD treatments. Sufficient light promoted grain filling and formation, thereby increasing grain weight, while shading negatively impacted grain filling rates (Kiniry and Ritchie, 1985; Setter et al., 2001; Jia et al., 2011). Nitrogen application significantly improved grain weight growth characteristics (Walter et al., 2003; Brouwer et al., 2012).

#### 4.2.3 Coordinated effects of planting density and nitrogen rate on source-sink relationships

The results indicated that, with increasing planting density, the correlation between sink capacity (SC) and grain yield gradually strengthened, whereas the correlation between source and grain yield progressively weakened (**Figure 9B**). Specifically, the coefficients of determination (R²) between sink capacity (grain numbers per year) and yield under LD, MD, and HD treatments were 0.235, 0.654, and 0.670, respectively, while those between source (maximum leaf area index) and yield were 0.221, 0.104, and 0.022, respectively. In combination with the quantitative source–sink indicators, we found that under high-density (HD) planting, source increased by 17.6% compared with low density (LD) and by 5.4% compared with medium density (MD). In contrast, sink increased by 15.2% relative to LD but decreased by 7.1% relative to MD. This suggests that, although both source and sink increase with planting density, the rate of increase in source exceeds that of sink. Consequently, under HD conditions, source supply becomes relatively excessive, whereas sink expansion is insufficient. As a result, yield formation becomes increasingly dependent on sink capacity, and sink limitation gradually emerges as the primary constraint to further yield improvement, which consistent with previous research (Ma et al., 2025).

Nitrogen application promoted increases in source growth (SG), sink growth (SC), and dry matter source supply rate (DSS) per unit of dry matter (**Table 4**). However, no significant differences were observed between N3 and N2 treatments. This was likely due to that excessive leaf growth reduced the allocation of photosynthetic products to sinks, thereby hindering sink capacity expansion (Lv et al., 2020). Insufficient sink capacity can also produce feedback inhibition, causing photosynthetic products to accumulate on the source side, thereby affecting leaf photosynthesis and rapid sink (grain) expansion (Li et al., 2020). Additionally, nitrogen deficiency reduced source supply capacity, leading to yield gaps, as reductions in source capacity directly resulted in fewer kernels per ear and reduced grain weight (Abeledo et al., 2020; Tian et al., 2020). Nitrogen application enhanced source supply rate (SUR) and sink growth rate (SGR), and changes in source capacity during the grain filling stage primarily resulted from increases in source growth (SG), thereby promoting sustained source-to-sink supply.

### 4.3 Maintaining post-silking leaf function and balanced source–sink relations to sustain maize yield and improve water productivity

Leaf senescence is intrinsically linked to source–sink coordination in cereal crops (Luo et al., 2018). Rather than examining individual management factors in isolation, we quantified the critical temporal relationship between the occurrence of the maximum grain-filling rate and the onset of post-silking ear-leaf functional decline. Our results showed that increasing planting density markedly shortened this interval (by 7.4–15.3 days), reflecting an earlier loss of effective source activity under intensified competition (**Table 5**). In contrast, appropriate nitrogen supply prolonged this interval by 1.02–5.77 days by alleviating stress-induced premature leaf functional decline. However, yield gains under high nitrogen input were not proportional to the extended duration of leaf greenness.

**Table 5:** The “time interval” indicates the difference gap between the date when the decay rate of the senescence index reached 95% of the theoretical value and the time of the maximum grain-filling rate.

Treatment	2020	2021
LDN0	3.39	2.7
LDN1	7.05	5.72
LDN2	9.84	8.09
LDN3	16.32	13.62
MDN0	0.2	-0.53
MDN1	0.13	0.51
MDN2	2.15	0.01
MDN3	4.43	0.63
HDN0	-8.86	-7.02
HDN1	-10.12	-7.29
HDN2	-5.53	-6.92
HDN3	-4.59	-5.92

Taken together, these findings challenge the traditional view that maximizing the duration of green leaf area is inherently beneficial for yield formation. Instead, our results demonstrate that the synchronization between the onset of leaf functional decline and the period of maximum grain filling is more critical than the absolute persistence of green leaves. While appropriate nitrogen application (N2) helped maintain leaf function during the critical grain-filling stage, excessive nitrogen input (N3) disrupted source–sink coordination by prolonging leaf functional maintenance beyond the optimal physiological window. This mismatch constrained the timely remobilization of stored assimilates from source tissues to developing grains, thereby weakening their contribution to final yield despite increased biomass accumulation. Therefore, high yield formation depends not on delaying senescence per se, but on optimizing the temporal alignment between peak grain filling and the natural progression of leaf functional decline to ensure efficient assimilate transfer. Furthermore, we found that under high planting density, the system undergoes a fundamental shift from source-limitation to sink-limitation. Specifically, the rate of source capacity expansion increased 17.6% under high density disproportionately outpaces sink growth increased 15.2%, creating a relative surplus of source supply against an insufficient sink. This imbalance leads to feedback inhibition, where accumulated photosynthetic products cannot be effectively utilized, restricting further yield potential.

In terms of resource efficiency, the D2N2 configuration also maximized WP and IWP by ensuring the senescence timing fell within an optimal range for converting transpired water into grain biomass. Therefore, the optimal D2N2 configuration identified here represents a physiology-based strategy that precisely regulates this source–sink ratio. It avoids the “sink-limitation trap” of high densities and the “remobilization inefficiency” of excessive nitrogen, achieving a superior trade-off among yield formation, water productivity, and nitrogen use efficiency.

The optimal planting density (medium density, MD: 100,000 plants ha-¹) and nitrogen application rate (N2: 180 kg N ha-¹) proposed in this study exhibit a certain degree of regional applicability. In the drip-irrigated maize production system of the arid Xinjiang region, Zhang reported that maximum yield was achieved at a substantially higher planting density of 120,000 plants ha-¹ combined with a nitrogen application rate of 360 kg N ha-¹, which clearly exceeds the recommendations of the present study (Zhang et al., 2025). This discrepancy is primarily attributable to pronounced differences in regional light and thermal resources: Xinjiang is characterized by prolonged sunshine duration (annual sunshine hours of 2,500–3,500 h) and a higher accumulated temperature (≥10 °C active accumulated temperature of 3,000–5,500 °C). Such ample light and heat conditions can support efficient light interception and conversion in high-density stands (Ren et al., 2022). Meanwhile, the drip irrigation system can precisely satisfy crop water requirements under high-density conditions, thereby alleviating water stress and reducing its constraints on nitrogen uptake (Ma et al., 2021).

## 5 Conclusions

Increasing nitrogen rate and planting density significantly reduced T_1_, GALD_1ear_, delayed t_0_, reduced V_max_ and increased post-silking dry matter translocation. Increasing nitrogen rate and planting density increased source growth (SG), sink capacity (SC) and difference of source sink (DSS), dry matter accumulation, both of which advanced the time of emergence of maximum dry matter growth rate, and increased the average and maximum dry matter growth rates (R_a_ and R_m_). Source growth (SG) under high-density planting was 5.5% and 19.6% lower than that under medium- and low-density planting, respectively. Maize sink capacity (SC) increased by 19.3% under low-density planting compared to medium-density planting. Nitrogen application increased the source supply rate (SUR) and sink growth rate (SGR) and increased the time interval; increased planting density decreased the source supply rate (SUR) and narrowed the time interval. Under low nitrogen planting, source limitation became an important factor limiting high grain yield; under high-density planting, sink limitation became an important reason limiting high grain yield. The D2N3 treatment demonstrated the highest grain yield, while D2N2 ranked second in yield but significantly enhanced water productivity (WP) and irrigation water productivity (IWP). Based on multi-objective trade-offs involving source-sink coordination optimization, high-efficiency water utilization, and yield stability, D2N2 was established as the optimal regional cultivation paradigm.

## Data Availability

The original contributions presented in the study are included in the article/**Supplementary Material**. Further inquiries can be directed to the corresponding author.

## References

[B1] Abeledo L. G. Savin R. Slafer G. A. (2020). Maize senescence under contrasting source-sink ratios during the grain filling period. Environ. Exp. Bot. 180, 104263. doi: 10.1016/j.envexpbot.2020.104263

[B2] Antonietta M. Fanello D. D. Acciaresi H. A. Guiamet J. J. (2014). Senescence and yield responses to plant density in stay green and earlier-senescing maize hybrids from Argentina. Field Crops Res. 155, 111–119. doi: 10.1016/j.fcr.2013.09.016

[B3] Arata A. F. Lázaro L. Tranquilli G. E. Arrigoni A. C. Martínez M. Rondanini D. P. (2023). How does post-flowering source/sink manipulation affect grain weight and commercial quality in Argentinean bread wheat genotypes with different baking aptitude? Field Crops Res. 301, 109030. doi: 10.1016/j.fcr.2023.109030

[B4] Borrás L. Slafer G. A. Otegui M. E. (2004). Seed dry weight response to source–sink manipulations in wheat, maize and soybean: a quantitative reappraisal. Field Crops Res. 86, 131–146. doi: 10.1016/j.fcr.2003.08.002

[B5] Brouwer B. Ziolkowska A. Bagard M. Keech O. Gardeström P. (2012). The impact of light intensity on shade-induced leaf senescence. Pl. Cell Environ. 35, 1084–1098. doi: 10.1111/j.1365-3040.2011.02474.x, PMID: 22171633

[B6] Cao Z.-Y. Chen Z.-H. Tang B. Zeng Q. Guo H.-L. Huang W.-H. . (2024). The effects of sowing date on maize: Phenology, morphology, and yield formation in a hot subtropical monsoon region. Field Crops Res. 309, 109309. doi: 10.1016/j.fcr.2024.109309

[B7] Causin H. F. Jauregui R. N. Barneix A. J. (2006). The effect of light spectral quality on leaf senescence and oxidative stress in wheat. Plant Sci. 171, 24–33. doi: 10.1016/j.plantsci.2006.02.009

[B8] Chan S. S. McCreight R. W. Walstad J. D. Spies T. A. (1986). Evaluating forest vegetative cover with computerized analysis of fisheye photographs. For. Sci. 32, 1085–1091. doi: 10.1093/forestscience/32.4.1085

[B9] Chen Y. Xiao C. Wu D. Xia T. Chen Q. Chen F. . (2015). Effects of nitrogen application rate on grain yield and grain nitrogen concentration in two maize hybrids with contrasting nitrogen remobilization efficiency. Eur. J. Agron. 62, 79–89. doi: 10.1016/j.eja.2014.09.008

[B10] Craine J. M. Dybzinski R. (2013). Mechanisms of plant competition for nutrients, water and light. Funct. Ecol. 27, 833–840. doi: 10.1111/1365-2435.12081

[B11] Distelfeld A. Avni R. Fischer A. M. (2014). Senescence, nutrient remobilization, and yield in wheat and barley. J. Exp. Bot. 65, 3783–3798. doi: 10.1093/jxb/ert477, PMID: 24470467

[B12] Dosio G. A. A. Cicore P. Rizzalli R. (2024). A time-window within the grain filling period accounted for early leaf senescence in maize under sink limitation. Eur. J. Agron. 152, 127027. doi: 10.1016/j.eja.2023.127027

[B13] Gregersen P. L. Culetic A. Boschian L. Krupinska K. (2013). Plant senescence and crop productivity. Plant Mol. Biol. 82, 603–622. doi: 10.1007/s11103-013-0013-8, PMID: 23354836

[B14] Havé M. Marmagne A. Chardon F. Masclaux-Daubresse C. (2017). Nitrogen remobilization during leaf senescence: lessons from Arabidopsis to crops. J. Exp. Bot. 68, 2513–2529. doi: 10.1093/jxb/erw365, PMID: 27707774

[B15] He P. Osaki M. Takebe M. Shinano T. (2002). Changes of photosynthetic characteristics in relation to leaf senescence in two maize hybrids with different senescent appearance. Photosynthetica 40, 547–552. doi: 10.1023/A:1024347918199

[B16] Heath R. L. Packer L. (1968). Photoperoxidation in isolated chloroplasts: I. Kinetics and stoichiometry of fatty acid peroxidation. Arch. Biochem. Biophys. 125, 189–198. doi: 10.1016/0003-9861(68)90654-1, PMID: 5655425

[B17] Jia S. Li C. Dong S. Zhang J. (2011). Effects of shading at different stages after anthesis on maize grain weight and quality at cytology level. Agric. Sci. China 10, 58–69. doi: 10.1016/S1671-2927(11)60307-6

[B18] Jia Q. Sun L. Mou H. Ali S. Liu D. Zhang Y. . (2018). Effects of planting patterns and sowing densities on grain-filling, radiation use efficiency and yield of maize (Zea mays L.) in semi-arid regions. Agric. Water Manage. 201, 287–298. doi: 10.1016/j.agwat.2017.11.025

[B19] Joshi S. Choukimath A. Isenegger D. Panozzo J. Spangenberg G. Kant S. (2019). Improved wheat growth and yield by delayed leaf senescence using developmentally regulated expression of a cytokinin biosynthesis gene. Front. Plant Sci. 10. doi: 10.3389/fpls.2019.01285, PMID: 31681380 PMC6813231

[B20] Kiniry J. R. Ritchie J. T. (1985). Shade-sensitive interval of kernel number of maize 1. Agron. J. 77, 711–715. doi: 10.2134/agronj1985.00021962007700050012x

[B21] Lai Z. Liao Z. Liu Y. Kou H. Li Z. Fan J. (2025). Optimized planting density and nitrogen rate improved grain yield of drip-fertigated maize by enhancing canopy structure and photosynthetic capacity. J. Agric. Food Res. 21, 101813. doi: 10.1016/j.jafr.2025.101813

[B22] Lan T. Du L. Wang X. Zhan X. Liu Q. Wei G. . (2024). Synergistic effects of planting density and nitrogen fertilization on chlorophyll degradation and leaf senescence after silking in maize. Crop J. 12, 605–613. doi: 10.1016/j.cj.2024.02.006

[B23] Li G. Cheng G. Li L. Lu D. Lu W. (2020). Effects of slow-released fertilizer on maize yield, biomass production, and source-sink ratio at different densities. J. Plant Nutr. 43, 725–738. doi: 10.1080/01904167.2019.1701027

[B24] Li Y. Ming B. Fan P. Liu Y. Wang K. Hou P. . (2022). Quantifying contributions of leaf area and longevity to leaf area duration under increased planting density and nitrogen input regimens during maize yield improvement. Field Crops Res. 283, 108551. doi: 10.1016/j.fcr.2022.108551

[B25] Li J. Xie R. Z. Wang K. R. Ming B. Guo Y. Q. Zhang G. Q. . (2015). Variations in maize dry matter, harvest index, and grain yield with plant density. Agron. J. 107, 829–834. doi: 10.2134/agronj14.0522

[B26] Li R. Zhang G. Liu G. Wang K. Xie R. Hou P. . (2021). Improving the yield potential in maize by constructing the ideal plant type and optimizing the maize canopy structure. Food Energy Secur. 10, e312. doi: 10.1002/fes3.312

[B27] Li X. Long A. Ji X. Wang X. Wang Z. Gong X. . (2024). Straw return and nitrogen fertilizer application regulate the efficient use of radiation, water, nitrogen and maize productivity in Northeast China. Agric. Water Manag. 301, 108973. doi: 10.1016/j.agwat.2024.108973

[B28] Liang X. Gao Z. Zhang L. Shen S. Zhao X. Liu Y. P. . (2019). Seasonal and diurnal patterns of non-structural carbohydrates in source and sink tissues in field maize. BMC Plant Biol. 19, 1–11. doi: 10.1186/s12870-019-2068-4, PMID: 31752685 PMC6868840

[B29] Liu Z. Gao J. Zhao S. Sha Y. Huang Y. Hao Z. . (2023). Nitrogen responsiveness of leaf growth, radiation use efficiency and grain yield of maize (Zea mays L.) in Northeast China. Field Crops Res. 291, 108806. doi: 10.1016/j.fcr.2022.108806

[B30] Liu G. Hou P. Xie R. Ming B. Wang K. Xu W. . (2017). Canopy characteristics of high-yield maize with yield potential of 22.5 Mg ha^–1^. Field Crops Res. 213, 221–230. doi: 10.1016/j.fcr.2017.08.011

[B31] Luo Z. Liu H. Li W. Zhao Q. Dai J. Tian L. . (2018). Effects of reduced nitrogen rate on cotton yield and nitrogen use efficiency as mediated by application mode or plant density. Field Crops Res. 218, 150–157. doi: 10.1016/j.fcr.2018.01.003

[B32] Lv X. Zhang Y. Zhang Y. Fan S. Kong L. (2020). Source-sink modifications affect leaf senescence and grain mass in wheat as revealed by proteomic analysis. BMC Plant Biol. 20, 257. doi: 10.1186/s12870-020-02447-8, PMID: 32503423 PMC7275590

[B33] Ma Y. Ren J. Yang S. Ding R. Du T. Kang S. . (2025). Enhancing maize yield and water productivity through coordinated root-shoot growth under mild water stress in dense planting. Field Crops Res. 323, 109786. doi: 10.1016/j.fcr.2025.109786

[B34] Ma L. Zhang X. Lei Q. Liu F. (2021). Effects of drip irrigation nitrogen coupling on dry matter accumulation and yield of Summer Maize in arid areas of China. Field Crops Res. 274, 108321. doi: 10.1016/j.fcr.2021.108321

[B35] Mason T. G. Maskell E. J. (1928). Studies on the transport of carbohydrates in the cotton plant: II. The Factors determining the Rate and the Direction of Movement of Sugars1. Ann. Bot. 42, 571–636. doi: 10.1093/oxfordjournals.aob.a090131

[B36] Mu X. Chen Y. (2021). The physiological response of photosynthesis to nitrogen deficiency. Plant Physiol. Biochem. 158, 76–82. doi: 10.1016/j.plaphy.2020.11.019, PMID: 33296848

[B37] Ning P. Fritschi F. B. Li C. (2017). Temporal dynamics of post-silking nitrogen fluxes and their effects on grain yield in maize under low to high nitrogen inputs. Field Crops Res. 204, 249–259. doi: 10.1016/j.fcr.2017.01.022

[B38] Ordóñez R. A. Savin R. Cossani C. M. Slafer G. A. (2018). Maize grain eeight sensitivity to source–sink manipulations under a wide range of field conditions. Crop Sci. 58, 2542–2557. doi: 10.2135/cropsci2017.11.0676

[B39] Ouyang Y. Evans S. E. Friesen M. L. Tiemann L. K. (2018). Effect of nitrogen fertilization on the abundance of nitrogen cycling genes in agricultural soils: A meta-analysis of field studies. Soil Biol. Biochem. 127, 71–78. doi: 10.1016/j.soilbio.2018.08.024

[B40] Ren H. Qi H. Zhao M. Zhou W. Wang X. Gong X. . (2022). Characterization of source–sink traits and carbon translocation in maize hybrids under high plant density. Agronomy 12, 961. doi: 10.3390/agronomy12040961

[B41] Setter T. L. Flannigan B. A. Melkonian J. (2001). Loss of kernel set due to water deficit and shade in maize. Crop Sci. 41, 1530–1540. doi: 10.2135/cropsci2001.4151530x

[B42] Shao L. Liu Z. Li H. Zhang Y. Dong M. Guo X. . (2021). The impact of global dimming on crop yields is determined by the source–sink imbalance of carbon during grain filling. Global Change Biol. 27, 689–708. doi: 10.1111/gcb.15453, PMID: 33216414

[B43] Thiex N. Van Erem T. (1999). Comparisons of karl fischer method with oven methods for determination of water in forages and animal feeds. J. AOAC. Int. 82, 799–808. doi: 10.1093/jaoac/82.4.799 9477559

[B44] Tian B. Zhu J. Liu X. Huang S. Wang P. (2020). Interacting leaf dynamics and environment to optimize maize sowing date in North China Plain. J. Integr. Agric. 19, 1227–1240. doi: 10.1016/S2095-3119(19)62831-5

[B45] Tokatlidis I. S. Has V. Melidis V. Has I. Mylonas I. Evgenidis G. . (2011). Maize hybrids less dependent on high plant densities improve resource-use efficiency in rainfed and irrigated conditions. Field Crops Res. 120, 345–351. doi: 10.1016/j.fcr.2010.11.006

[B46] Van Oosterom E. J. Jayachandran R. Bidinger F. R. (1996). Diallel analysis of the stay-green trait and its components in sorghum. Crop Sci. 36, 549–555. doi: 10.2135/cropsci1996.0011183X003600030002x. cropsci1996.0011183X003600030002x.

[B47] Walter A. Roggatz U. Schurr U. (2003). Expansion kinematics are an intrinsic property of leaf development and are scaled from cell to leaf level at different nutrient availabilities. Plant Biol. 5, 642–650. doi: 10.1055/s-2003-44691

[B48] Wang W. Li M.-Y. Zhou R. Mo F. Khan A. Batool A. . (2023). Leaf senescence, nitrogen remobilization, and productivity of maize in two semiarid intercropping systems. Eur. J. Agron. 150, 126943. doi: 10.1016/j.eja.2023.126943

[B49] Wang X. Ye T. Ata-Ul-Karim S. T. Zhu Y. Liu L. Cao W. . (2017). Development of a critical nitrogen dilution curve based on leaf area duration in Wheat. Front. Plant Sci. 8. doi: 10.3389/fpls.2017.01517, PMID: 28928757 PMC5591374

[B50] Wei J. Chai Q. Yin W. Fan H. Guo Y. Hu F. . (2024). Grain yield and N uptake of maize in response to increased plant density under reduced water and nitrogen supply conditions. J. Integr. Agric. 23, 122–140. doi: 10.1016/j.jia.2023.05.006

[B51] Wu X. Tong L. Kang S. Du T. Ding R. Li S. . (2023). Combination of suitable planting density and nitrogen rate for high yield maize and their source–sink relationship in Northwest China. J. Sci. Food Agric. 103, 5300–5311. doi: 10.1002/jsfa.12602, PMID: 37016583

[B52] Wu Y. Zhao B. Li X. Liu Q. Feng D. Lan T. . (2022). Nitrogen application affects maize grain filling by regulating grain water relations. J. Integr. Agric. 21, 977–994. doi: 10.1016/S2095-3119(20)63589-4

[B53] Xu L. Zheng C. Ma Y. (2021). Variations in precipitation extremes in the arid and semi-arid regions of China. Int. J. Climatol. 41, 1542–1554. doi: 10.1002/joc.6884

[B54] Yan S. Wu Y. Fan J. Zhang F. Zheng J. Guo J. . (2022). Source-sink relationship and yield stability of two maize cultivars in response to water and fertilizer inputs in northwest China. Agric. Water Manage. 262, 107332. doi: 10.1016/j.agwat.2021.107332

[B55] Yin X. Goudriaan J. Lantinga E. A. Vos J. Spiertz H. J. (2003). A flexible sigmoid function of determinate growth. Ann. Bot. 91, 361–371. doi: 10.1093/aob/mcg029, PMID: 12547689 PMC4244967

[B56] Yin X. Guo W. Spiertz J. H. (2009). A quantitative approach to characterize sink–source relationships during grain filling in contrasting wheat genotypes. Field Crops Res. 114, 119–126. doi: 10.1016/j.fcr.2009.07.013

[B57] Zhang L.-X. Li S.-X. Zhang H. Liang Z.-S. (2007). Nitrogen rates and water stress effects on production, lipid peroxidation and antioxidative enzyme activities in two maize (Zea mays L.) genotypes. J. Agron. Crop Sci. 193, 387–397. doi: 10.1111/j.1439-037X.2007.00276.x

[B58] Zhang Y. Zhai J. Zhang G. Cao Y. Xu W. Ming B. . (2025). Differential nitrogen demand in maize under high and low planting densities. J. Agric. Food Res. 24, 102446. doi: 10.1016/j.jafr.2025.102446

