# Supplementary material for: Optimized planting density and nitrogen improve grain yield and water productivity in drip-fertigated maize through improved canopy function and source–sink balance
Source: Front Plant Sci. 2026 Feb 19;17:1740500. doi: 10.3389/fpls.2026.1740500 (PMC12963823; doi:10.3389/fpls.2026.1740500)
Supplement: Supplementary file 1 [file SupplementaryFile1.docx]

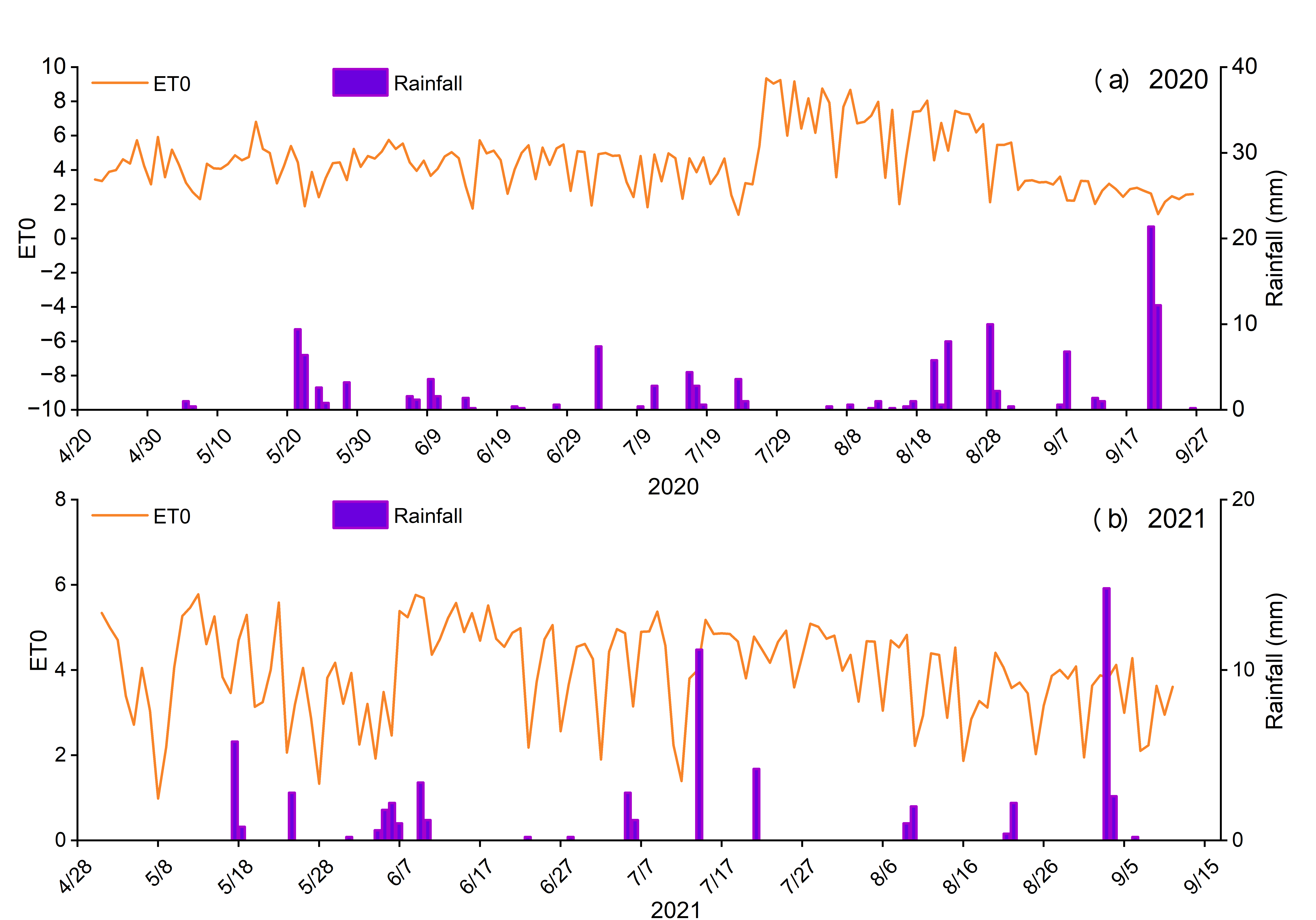


**Fig. S1** Daily Reference evapotranspiration (ET_0_) and precipitation during 2020 and 2021.


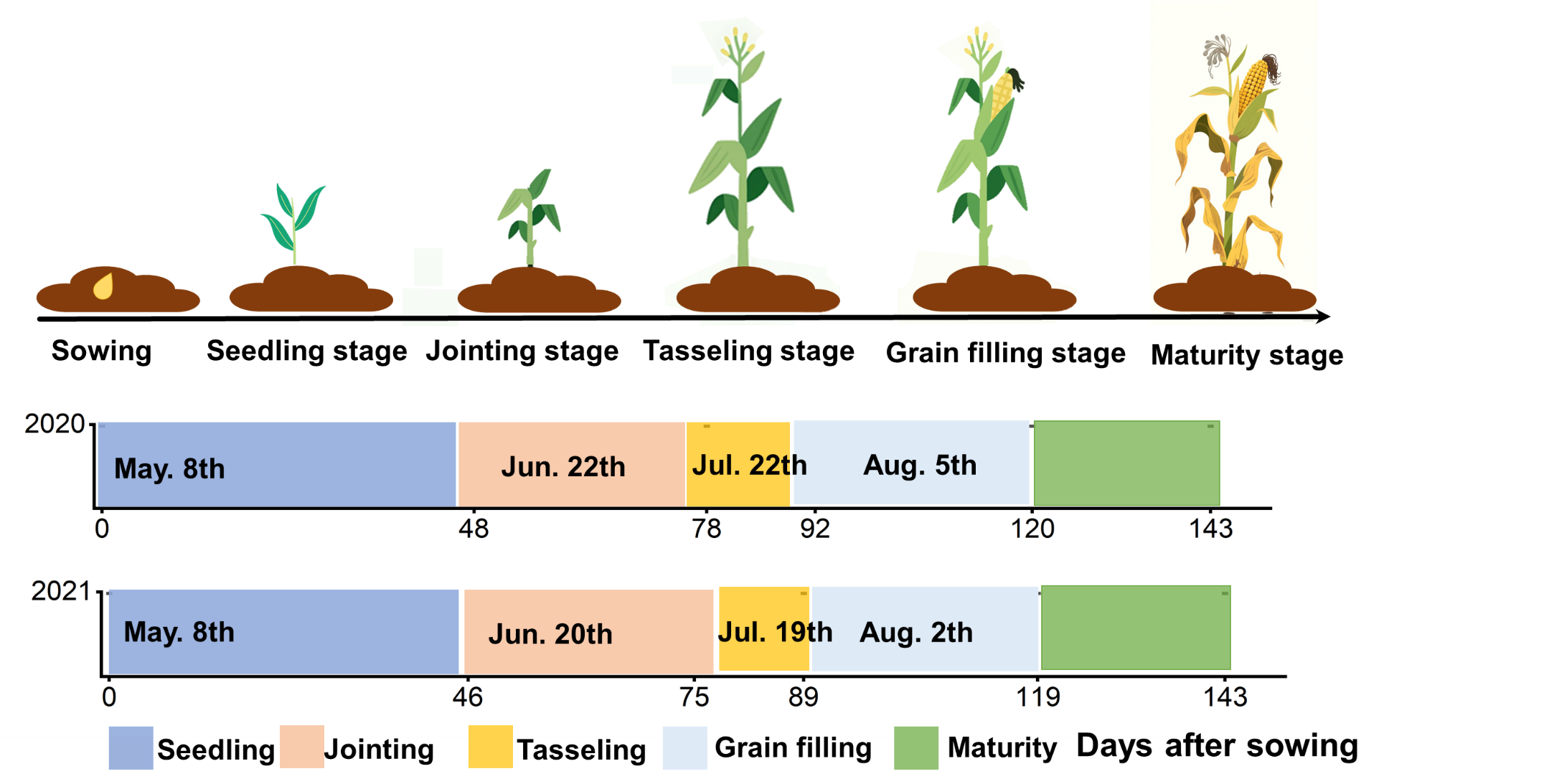


**Fig.** **S2** Maize growth periods in 2020 and 2021.

**Table S1** The values of Cronbach's Alpha, rho_A, CR and AVE in PLS-SEM.

|  | Cronbach's Alpha | rho_A | CR | AVE |
| --- | --- | --- | --- | --- |
| Planting density | 1.000 | 1.000 | 1.000 | 1.000 |
| Nitrogen rate | 1.000 | 1.000 | 1.000 | 1.000 |
| LAD | 0.948 | 0.947 | 0.957 | 0.867 |
| Leaf senescence parameters | 0.792 | 0.786 | 0.821 | 0.747 |
| Source growth parameters | 0.842 | 0.782 | 0.812 | 0.903 |
| Source-sink parameters | 0.882 | 0.864 | 0.914 | 0.785 |
| Grain yield components | 0.799 | 0.851 | 0.855 | 0.796 |
| Grain yield | 0.894 | 0.778 | 0.811 | 0.913 |

| 2021 | a | b | c | R^2^ | t_0_ | T_1_ | V_1_ | GLAD1_ear_ | T_2_ | V_2_ | GLAD2_ear_ | V_max_ |
| --- | --- | --- | --- | --- | --- | --- | --- | --- | --- | --- | --- | --- |
| LDN0 | 798.41b | -0.081b | 43.53c | 0.996 | 24.35b | 40.31c | -3.58b | 25978.99c | 25.82c | -15.28c | 8846.17c | -17.43c |
| LDN120 | 874.72b | -0.082b | 46.39b | 0.997 | 26.24ab | 44.13b | -3.54b | 30724.17b | 28.94b | -14.75b | 10731.45b | -16.83b |
| LDN180 | 971.38ab | -0.091ab | 49.12ab | 0.997 | 27.92a | 47.77a | -3.59ab | 36469.47ab | 32.12a | -14.59ab | 13033.04a | -16.64a |
| LDN240 | 1074.71a | -0.102a | 52.03a | 0.999 | 32.25a | 52.34a | -3.28a | 40146.21a | 32.52a | -14.41a | 13198.89a | -16.44a |
| **AV.** | **929.81A** | **-0.089A** | **47.77A** |  | **27.69A** | **46.14A** | **-3.4975A** | **33329.71A** | **29.85A** | **-14.76AB** | **11452.3875A** | **-16.835A** |
| MDN0 | 799.78b | -0.072c | 39.41c | 0.994 | 21.36a | 33.32b | -4.41c | 21639.82c | 25.82d | -15.56d | 8959.66c | -17.74c |
| MDN120 | 849.07b | -0.081b | 41.95b | 0.991 | 22.41a | 35.5b | -4.34b | 24110.22b | 29.27c | -14.4c | 10623.9b | -16.42b |
| MDN180 | 993.91aa | -0.09ab | 45.1a | 0.983 | 22.85a | 36.94ab | -4.37b | 26076.04ab | 32.52b | -13.57b | 12471.56ab | -15.47a |
| MDN240 | 1049.4a | -0.102a | 48.11a | 0.993 | 23.6a | 40.2a | -4.23a | 29898.72a | 36.58a | -12.71a | 14690.64a | -14.49a |
| **AV.** | **923.04A** | **-0.08625A** | **43.64B** |  | **22.56B** | **36.49B** | **-4.3375B** | **25431.2B** | **31.05A** | **-14.06A** | **11686.44A** | **-16.03A** |
| HDN0 | 712.58c | -0.086d | 37.52c | 0.971 | 19.39a | 32.73b | -4.25a | 20293.79c | 21.59c | -17.6d | 7088.59c | -20.07d |
| HDN120 | 802.2b | -0.092c | 39.95b | 0.983 | 19.14a | 34.5b | -4.24a | 22404.72b | 24.85b | -16.1c | 8590.47b | -18.36c |
| HDN180 | 893.01a | -0.106b | 42.14ab | 0.987 | 18.34a | 36.03ab | -4.43a | 25311.74a | 28.63a | -15.21b | 10793.3a | -17.35b |
| HDN240 | 1019.72a | -0.122a | 44.9a | 0.987 | 19.01a | 37.94a | -4.35a | 27531.24a | 30.63a | -14.72a | 11993.86a | -16.79a |
| **AV.** | **856.88B** | **-0.1015B** | **41.13B** |  | **18.97B** | **35.3B** | **-4.3175B** | **23885.3725B** | **26.425B** | **-15.98B** | **9616.56B** | **-18.14B** |
| F-value |  |  |  |  |  |  |  |  |  |  |  |  |
| D | ns | * | ** |  | ns | ** | ** | ** | ** | ** | ** | ** |
| N | ** | ** | ** |  | ns | ** | * | ** | ** | ** | ** | ** |
| D×N | ns | ns | ns |  | ns | ns | ns | ns | ns | ns | ns | ns |

**Table S**2. The logistic equation and its parameters for the dynamic change of ear leaf green area after silking in 2021.

a signifies the theoretical initial value of GLA_ear_, b is a parameter characterizing the rate of leaf functional decline, and c indicates the time at which the maximum rate of decline occurs. t_0_ is the time when the GLA_ear_ decreased to 95% of the theoretical initial value (d), T_1_ and T_2_ are the duration of the initial phase of leaf functional decline, V_1_ the corresponding rate of decline, and GLAD1_ear_ the cumulative green leaf area during this phase. Similarly, T₂ denotes the duration of the rapid functional decline phase, V_2_ the rapid decline rate, and GLAD2_ear_ the associated green leaf area duration. V_max_ denotes this maximum rate of leaf functional decline. Different lowercase letters to the right of the value indicate differences among treatments at the same density (p < 0.05). Different uppercase letters to the right of the value indicate differences among density treatments (p < 0.05). *, p <0.05; **, p <0.01. ns, not significant
